# Nuclear plasticity increases susceptibility to damage during confined migration

**DOI:** 10.1371/journal.pcbi.1008300

**Published:** 2020-10-09

**Authors:** Abhishek Mukherjee, Amlan Barai, Ramesh K. Singh, Wenyi Yan, Shamik Sen

**Affiliations:** 1 IITB-Monash Research Academy, IIT Bombay, Mumbai, India; 2 Dept. of Mechanical Engineering, IIT Bombay, Mumbai, India; 3 Dept. of Mechanical and Aerospace Engineering, Monash University, Melbourne, Australia; 4 Dept. of Biosciences & Bioengineering, IIT Bombay, Mumbai, India; University of Pennsylvania, UNITED STATES

## Abstract

Large nuclear deformations during migration through confined spaces have been associated with nuclear membrane rupture and DNA damage. However, the stresses associated with nuclear damage remain unclear. Here, using a quasi-static plane strain finite element model, we map evolution of nuclear shape and stresses during confined migration of a cell through a deformable matrix. Plastic deformation of the nucleus observed for a cell with stiff nucleus transiting through a stiffer matrix lowered nuclear stresses, but also led to kinking of the nuclear membrane. In line with model predictions, transwell migration experiments with fibrosarcoma cells showed that while nuclear softening increased invasiveness, nuclear stiffening led to plastic deformation and higher levels of DNA damage. In addition to highlighting the advantage of nuclear softening during confined migration, our results suggest that plastic deformations of the nucleus during transit through stiff tissues may lead to bending-induced nuclear membrane disruption and subsequent DNA damage.

## Author summary

Stiffness of the nucleus is known to impede migration of cells through dense matrices. Nuclear translocation through small pores is achieved by active deformation of the nucleus by the cytoskeleton. However, stresses on the nucleus during confined migration may lead to nuclear damage, as observed experimentally. However, the factors contributing to nuclear damage remain incompletely understood. Here we show that plastic or permanent nuclear deformation which is necessary for successful migration through small pores in stiff matrices, also leads to bending of the nuclear membrane. We propose that this bending precedes nuclear blebs which are experimentally observed.

## Introduction

Cells transit through a myriad of environments, ranging from 2D basement membranes (BM) to 3D collagen networks for morphogenesis, division and proliferation, wound healing and cancer invasion [1]. Cells sense the surrounding mechanical environment to decide on transiting through a pore, a decision that is intrinsically linked to its chances of survival [2]. PDMS devices, widely used for studying confined migration, are significantly stiffer (≈ MPa) than soft tissues (≈ kPa) *in vivo*, thus failing to recapitulate the interplay of nucleus and tissue properties that likely dictates the dynamics of confined migration. The importance of nuclear properties, namely stiffness, in regulating the efficiency of confined migration, is well appreciated. While physical properties of the nucleus are dictated by expression of the intermediate filament protein Lamin (A/C and B) [3–5] and its phosphorylation [6, 7], nuclear deformation is mediated by the actomyosin and the microtubule cytoskeleton which are physically coupled to the nucleus via nesprins [8, 9]. Additionally, localized cytoplasmic stiffening at sites of increased stress from the external environment [10–12], might facilitate nuclear compression thereby aiding in confined migration.

Computational modeling of cell migration has primarily been achieved either by idealizing them as solid continuum spring/spring-dashpot models [13–15] or as liquid droplets bounded by deformable membranes [16, 17]. These assumptions are reasonable in light of a cell being biphasic, exhibiting solid-like behaviour in certain situations and liquid-like character in others. However, both of these types of models have their limitations; whereas solid continuum models are unable to replicate similar levels of extreme cellular deformation that occurs in-vivo, liquid droplet models are unable to quantify intracellular stresses. Appropriate visualization of the evolution of stresses within cellular structures such as the actin cytoskeleton and nucleus is critical to complement experimental observations. The current state-of-the-art experimental procedures are unable to predict the stresses that the nucleus undergoes while migrating through 3D confined environments. Traction force microscopy that calculates the stress on the surface of a substrate by relating the deformation of that surface to the stress using Hooke’s law is a critical tool for visualizing mechanical interaction between a cell and its environment, but is limited by its inability to quantify intracellular stresses.

Extreme nuclear deformations during migration through micro-channels or pores have been shown to cause plastic deformation [18, 19] as well as nuclear membrane rupture [18–23]. Whether or not nuclear plasticity and nuclear damage are inter-related remains unknown. Also, the extent to which tissue properties influence the plastic deformation of the nucleus has not been probed. For probing nuclear deformation and deformation-induced damage, here we have developed a plane strain finite element model to simulate confined cell migration through a tissue-mimetic environment where mechanical properties of the cell and nucleus have been considered. Studying the collective influence of nuclear and tissue stiffness on the dynamics of pore migration, our results predict the magnitude of cellular force required to squeeze through a constriction and the intracellular stresses sustained by the cell. Our results predict that stiff nuclei passing through stiffer tissues undergo plastic deformations leading to nuclear membrane bending, which may be the cause of nuclear rupture documented experimentally. We validate these predictions using experiments wherein nuclear stiffening led to plastic deformation of the nucleus and higher DNA damage. In addition to predicting a scaling relationship between the timescales and force-scales associated with pore entry, our results establish a direct link between nuclear plasticity and nuclear damage during constricted migration.

## Results

### Nuclear and tissue properties collectively dictate dynamics of confined migration

The nucleus which is the largest and stiffest organelle inside the cell, is physically connected to the cytoskeleton through the LINC complex [24]. Consequently, compression of the cell during confined migration is associated with compression of the nucleus with the extent of cytoplasmic/nuclear deformations dictated by their mechanical properties in relation to that of the surrounding tissues. For studying dynamics of confined migration, a finite element model was developed wherein physical properties of cell membrane, cell cytoplasm, and nucleus were taken into account. Consistent with experiments, the cell membrane, cell cytoplasm and nuclear membrane were modeled as viscoelastic Kelvin-Voigt materials (S1 Fig) [25–27]. A similar viscoelastic description was also used in modeling tissue behavior [28]. Furthermore, consistent with stress-induced permanent deformation of the nucleus, an elastoplastic behavior was assumed for the nucleus [29, 30]. Finally, cytoskeletal strain stiffening behavior observed with reconstituted cytoskeletal networks was also accounted for [31–33].

**Fig 1:**
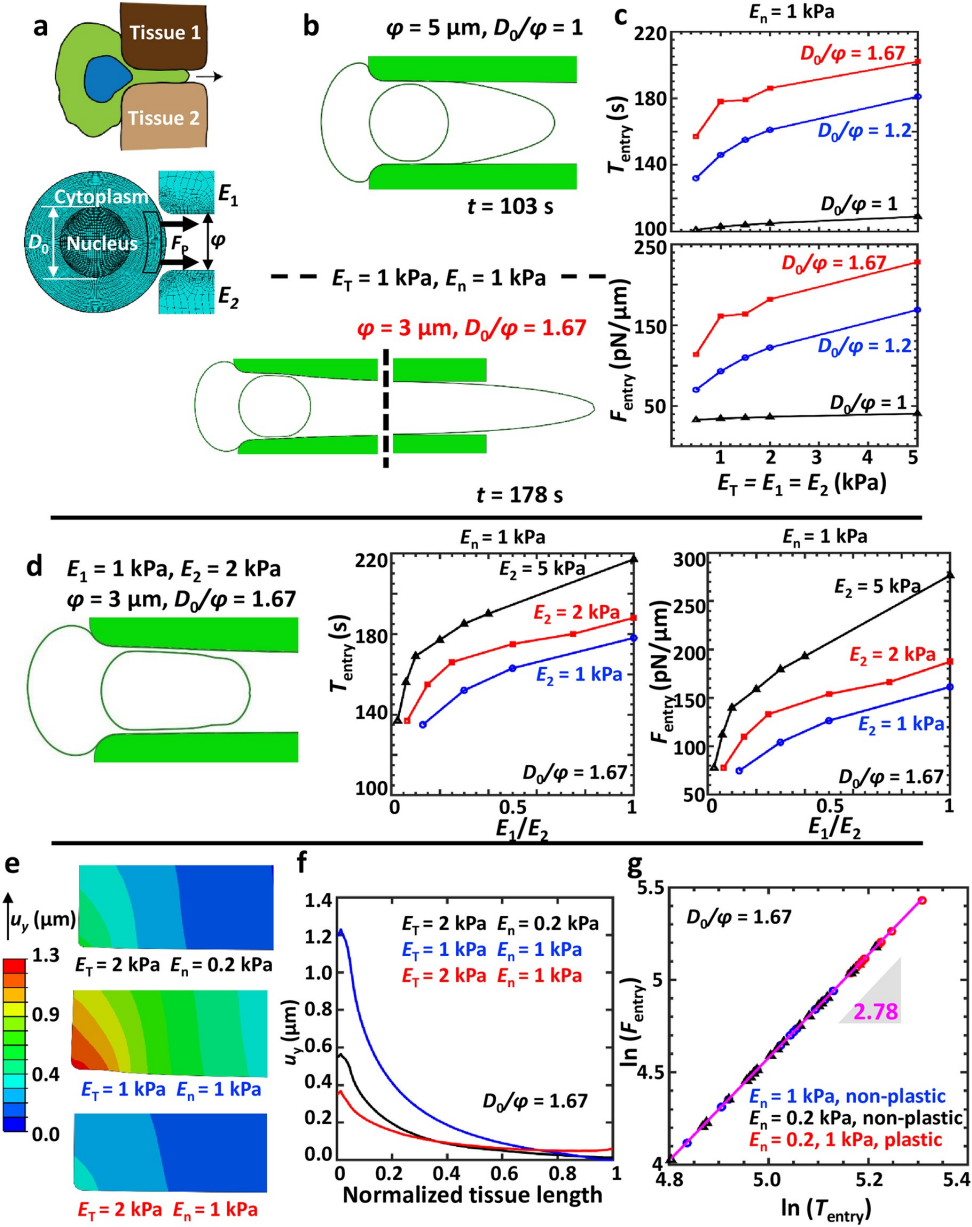
Interplay of nuclear and tissue stiffness on dynamics of pore entry. (a) Schematic of a cell squeezing through a pore in a given tissue or at the interface of two different tissues. *D*_0_ and *ϕ* correspond to the undeformed nucleus diameter and the undeformed pore diameter, respectively. *E*_1_ and *E*_2_ correspond to the stiffness of Tissue 1 and Tissue 2, respectively. (b) Cellular deformation just after entry into pore for different extents of degree of confinement (*D*_0_/*ϕ*). *E*_*c*_ was increased from an initial value of 1 Pa to a possible maximum of 1.1 Pa under shear-induced cytoskeletal stiffening and *E*_*n*_ was assumed to be 1 kPa. (c) Force (*F*_entry_) and time (*T*_entry_) required for a cell (with *E*_*n*_ = 1 kPa) to enter a pore of given size and their dependence on tissue stiffness (*E*_*T*_ = *E*_1_ = *E*_2_) and *D*_0_/*ϕ*. (d) Nuclear deformation for the case of cell entry through an interface between two dissimilar tissues. Dependence of *F*_entry_ and *T*_entry_ on *E*_1_/*E*_2_ for *D*_0_/*ϕ* = 1.67 and *E*_*n*_ = 1 kPa. (e) Contour plots of vertical tissue displacement (*u*_*y*_) at the time of nucleus entry into the pore, i.e., when the entire nucleus has just completed entering the pore. (f) Spatial dependence of *u*_*y*_ along the tissue length at the time of pore entry for different values of *E*_*T*_ and *E*_*n*_ and *D*_0_/*ϕ* = 1.67. Pore entry occurs at normalized tissue length = 0. (g) Scaling relationship between *F*_entry_ (pN/*μ*m) and *T*_entry_ (s) for *D*_0_/*ϕ* = 1.67.

In our model, cell migration through pores in tissues was assumed to be frictionless and mediated by protrusive forces (*F*_*P*_) exerted at the leading edge, with *E*_1_ and *E*_2_ representing the Young’s moduli (stiffness) of Tissue 1 and Tissue 2, respectively (Fig 1a). Simulation with *E*_1_ = *E*_2_ correspond to a situation wherein a cell squeezes through a pore in a given tissue/hydrogel. In comparison, *E*_1_ ≠ *E*_2_ corresponds to a cell migrating at the interface of two distinct tissues/hydrogels [34, 35]. To first probe the effect of nuclear size on migration efficiency, simulations were performed wherein dynamics of cell entry into a pore of given size (i.e., *ϕ* = {3, 5} *μ*m) was tracked for different sizes of nucleus (i.e., *D*_0_ = 5 and 6 *μ*m) and for varying tissue stiffness (i.e., *E*_*T*_: (0.13 − 5) kPa) (Fig 1b). In these simulations, nuclear stiffness was kept constant at *E*_*n*_ = 1 kPa. For entry into a pore within the same tissue, i.e., *E*_*T*_ = *E*_1_ = *E*_2_, the time for pore entry (*T*_entry_) as well as the maximum force required for pore entry (*F*_entry_) remained unchanged irrespective of *E*_*T*_ when the nucleus was smaller or equal to the pore size (i.e., *D*_0_/*ϕ* ≤ 1) (Fig 1c). However, both these quantities increased with increase in *E*_*T*_ for *D*_0_/*ϕ* > 1, highlighting the role of the nucleus in regulating confined migration. When *E*_1_ ≠ *E*_2_, *T*_entry_ and *F*_entry_ were comparable to values corresponding to the higher tissue stiffness (Fig 1d).

Entry into small pores (*D*_0_/*ϕ* = 1.67) was mediated by widening of the pores as evident from the vertical displacement of the tissues in a *E*_*n*_-dependent manner (Fig 1e). While displacements far from the pore entry decayed to zero in most cases, for the case corresponding to *E*_*T*_ = 2 kPa, *E*_*n*_ = 1 kPa, vertical displacement of the tissue was non-zero even at distances far from the entry point. The maximum vertical tissue displacement exhibited a non-monotonic dependence on *E*_*n*_/*E*_*T*_ with lowest displacement corresponding to *E*_*T*_ = 2 kPa, *E*_*n*_ = 1 kPa where non-zero displacements were observed far from the entry point (Fig 1f). Plotting of *F*_entry_ versus *T*_entry_ corresponding to *D*_0_/*ϕ* = 1.67 for different combinations of *E*_*T*_ and *E*_*n*_ revealed a nearly cubic scaling relationship with a factor of 2.78 (Fig 1g). Together, these results suggest that pore migration through deformable matrices is collectively dictated by nucleus and tissue properties with entry time-scales and force-scales strongly coupled to each other.

### Degree of confinement and nuclear/tissue properties collectively dictate average cell speed

**Fig 2:**
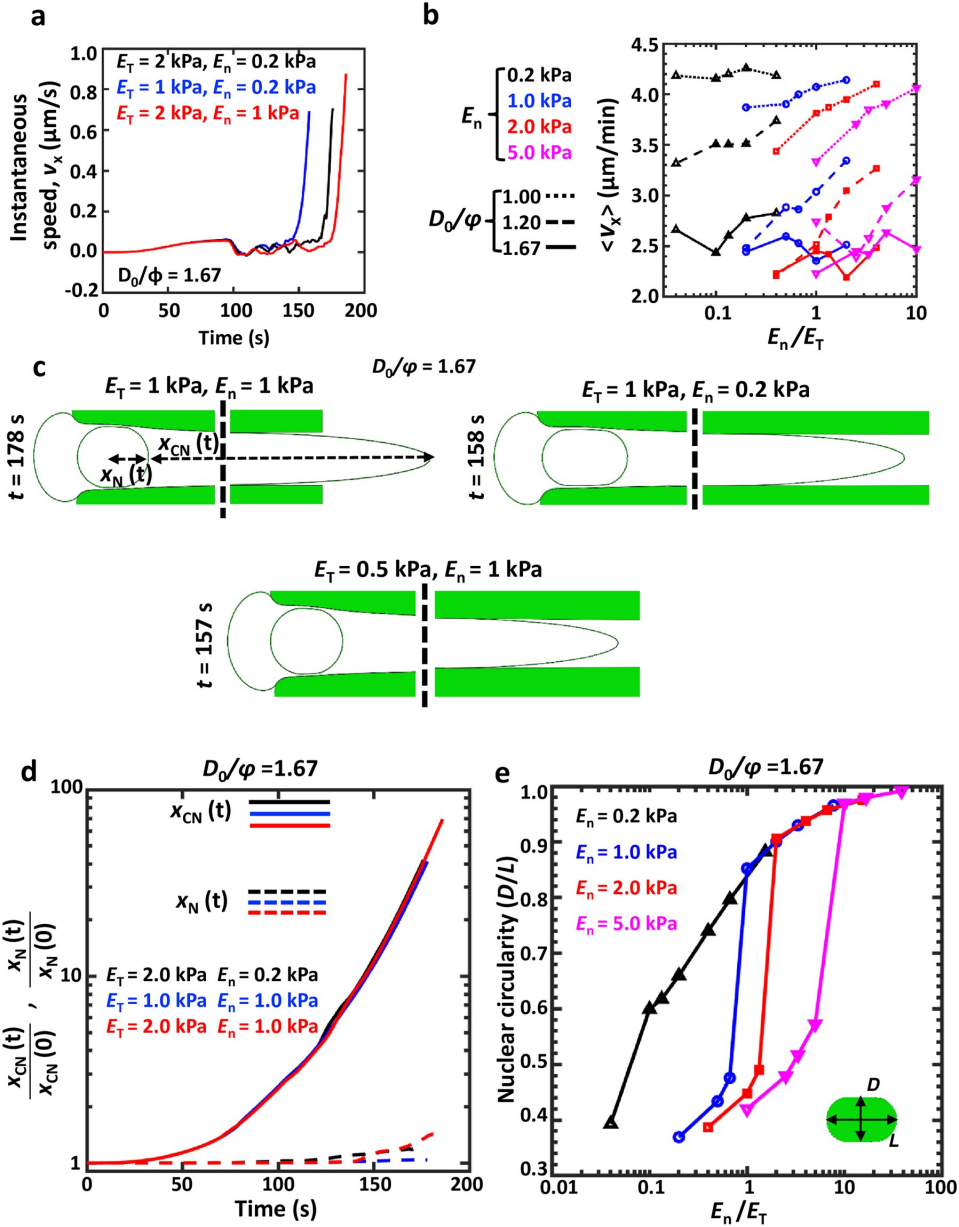
Morphological changes in cell/nucleus during confined migration. (a) Instantaneous cell velocity (*v*_*x*_) calculated from the start of the simulation (*t* = 0 s) till the instant of pore entry. (b) The dependence of average cell velocity (〈*v*_*x*_〉) on *E*_*n*_/*E*_*T*_ for different values of *E*_*n*_ and *D*_0_/*ϕ*. (c) Shapes of the cell and the nucleus at the time of pore entry for different combinations of *E*_*T*_ and *E*_*n*_ and *D*_0_/*ϕ* = 1.67. *x*_*CN*_(*t*) represents the distance between the leading edge of the cell and the front edge of the nucleus at time *t*. *x*_*N*_(*t*) represents the distance between the nucleus center and its front edge at time *t*. Dotted lines depict breaks in the cell profiles. (d) Temporal evolution of cytoplasmic stretch (*x*_*CN*_(*t*)/*x*_*CN*_(0)) and nuclear stretch (*x*_*N*_(*t*)/*x*_*N*_(0)) along the direction of migration for *D*_0_/*ϕ* = 1.67. (e) Dependence of nuclear circularity (*D*/*L*) on *E*_*n*_/*E*_*T*_ for *D*_0_/*ϕ* = 1.67 and different values of *E*_*n*_.

To probe how nuclear/tissue properties and the extent of confinement influence cell motility, cell velocity (*v*_*x*_) was tracked along the direction of migration, i.e., *x*-direction. *v*_*x*_ remained nearly zero for an extended duration, and shot up drastically towards the end (Fig 2a). The dependence of the average velocity 〈*v*_*x*_〉 on *E*_*n*_/*E*_*T*_ was dictated by *D*_0_/*ϕ* and *E*_*n*_ (Fig 2b). Sensitivity of 〈*v*_*x*_〉 to *E*_*n*_/*E*_*T*_ increased with *E*_*n*_ for all *D*_0_/*ϕ*. However, for *D*_0_/*ϕ* = 1.2, 〈*v*_*x*_〉 scaled positively with *E*_*n*_/*E*_*T*_ and negatively with *E*_*n*_.

Tracking temporal evolution of the normalized distance between the leading edge of the cell and the proximal edge of the nucleus (*x*_*CN*_(*t*)) revealed several-fold increase in *x*_*CN*_ over the initial undeformed distance *x*_*CN*_(0), indicative of cytoplasmic stretching in the direction of migration (Fig 2c and 2d). In comparison, the extent of nuclear stretch (*x*_*N*_(*t*)/*x*_*N*_(0)) was much less. While the period of near zero velocity coincided with duration of cytoplasmic stretch with negligible nuclear deformation, the sudden increase in cell velocity (*t* ≈ (150 − 180) sec) corresponded to nuclear entry into the pore. Nuclear circularity (i.e., *D*/*L*) plotted as a function of *E*_*n*_/*E*_*T*_ collapsed onto a master curve depending on the magnitude of *E*_*n*_ (Fig 2e). Lowest *D*/*L* (≈ 0.36) was observed for *E*_*n*_ = 1 kPa and *E*_*T*_ = 5 kPa. Together, these results suggest that cell speed is dictated not only by nuclear/tissue properties, but also by the extent of confinement.

### Plastic deformation of the nucleus and kink formation during pore entry

**Fig 3:**
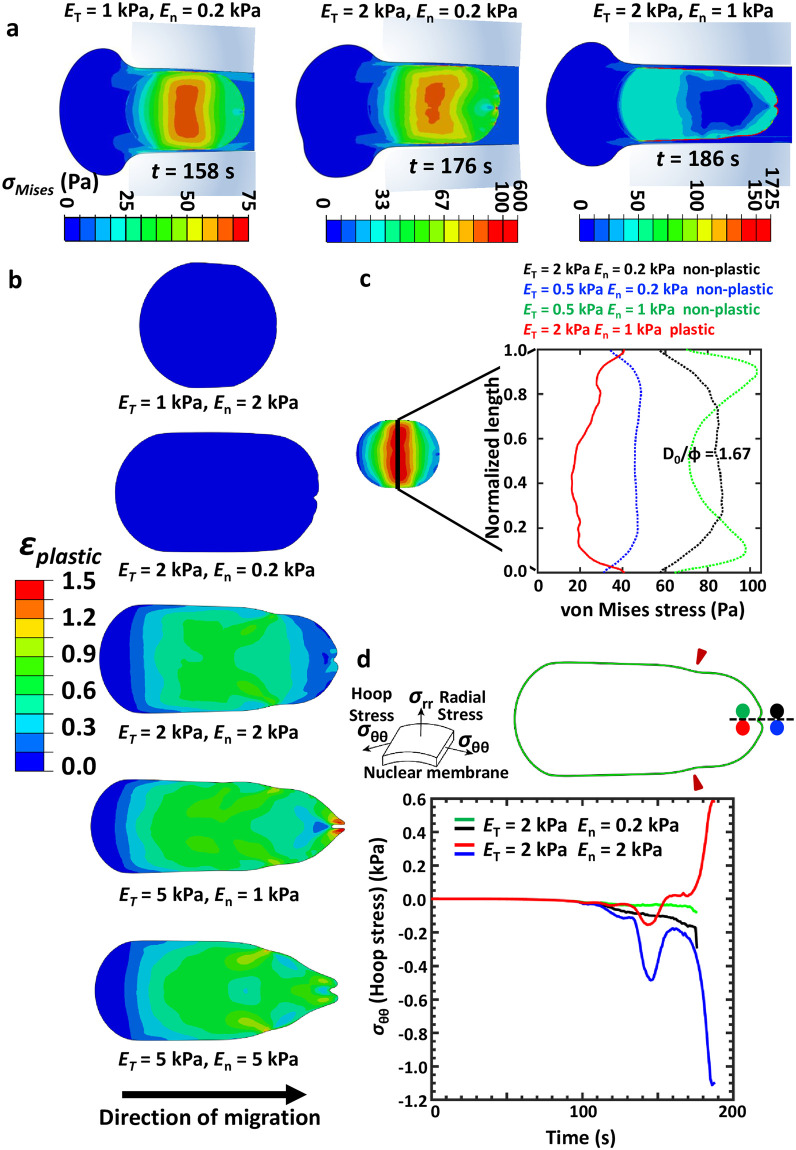
Nuclear plasticity during confined migration. (a) The spatiotemporal evolution of stress distribution just after entry of the 5*μ*m nucleus into a 3*μ*m pore, i.e., *D*_0_/*ϕ* = 1.67. Contours and colourbars indicate von Mises stresses (*σ*_*Mises*_) developed in the cytoplasm and nucleus, where $\sigma_{Mises}=\sqrt{\frac{1}{2}[{\left(\sigma_{xx}-\sigma_{yy}\right)}^{2}+{\left(\sigma_{yy}-\sigma_{zz}\right)}^{2}+{\left(\sigma_{zz}-\sigma_{xx}\right)}^{2}]+3(\tau_{xy}^{2}+\tau_{yz}^{2}+\tau_{zx}^{2})}$. Here, *σ*_*xx*_, *σ*_*yy*_ and *σ*_*zz*_ are normal stresses in the *x*, *y* and *z* directions in a Cartesian coordinate system and *τ*_*xy*_, *τ*_*yz*_ and *τ*_*zx*_ represent the shear stresses. (b) Spatial map of plastic strain (*ε*_*plastic*_) accumulated in the nucleus just after pore entry. The total strain (*ε*_*total*_) in a body is defined as the sum of elastic (*ε*_*elastic*_) and plastic strain, i.e., *ε*_*total*_ = *ε*_*elastic*_ + *ε*_*plastic*_. *ε*_*elastic*_ is defined as the reversible strain in the body whereas, *ε*_*plastic*_ is irreversible. We use a strain hardening material property definition given by: $\sigma =a+b\varepsilon_{plastic}^{n}$, where *σ* is the applied stress, *σ*_*yield*_ = *a*, and *a*, *b* and *n* are material properties. (c) Spatial distribution of von Mises stress in the nucleus along the vertical direction just after nuclear entry (*D*_0_/*ϕ* = 1.67). (d) Temporal evolution of hoop stresses (*σ*_*θθ*_) in the nuclear membrane from the start of simulation to the instant the nucleus completely enters the pore. The two cylindrical components of stresses, namely, radial (*σ*_*rr*_) and hoop (*σ*_*θθ*_) stress in the nuclear membrane are depicted along with the region of nuclear membrane from which the curves are extracted (*D*_0_/*ϕ* = 1.67). Green-Black and Red-Blue curves correspond to two different combinations of *E*_*T*_ and *E*_*n*_ as shown. Green and Red dots in the representative snapshot of the nuclear membrane correspond to kinked mesh elements on the nuclear membrane at its interface with the nucleus for *E*_*n*_ = 0.2 kPa and *E*_*n*_ = 2 kPa respectively. Similarly, Black and Blue dots correspond to kinked mesh elements on the nuclear membrane at its interface with the cytoplasm.

Alteration in nuclear circularity during pore entry is indicative of varying extents of nuclear stresses during and after entry (Fig 3a, S2 Fig). Among the representative cases shown in Fig 3a, the highest stress in the nucleus was observed for the case of *E*_*n*_ = 0.2 kPa, *E*_*T*_ = 2 kPa, where |*E*_*T*_ − *E*_*n*_| is maximum. Surprisingly, when the nucleus was 5 times stiffer (i.e., *E*_*n*_ = 1 kPa), stress in the nucleus was lower, and the nucleus was more elongated, raising the possibility of its plastic deformation. In our model, plastic deformation of the nucleus follows a strain hardening power law with the nuclear stress *σ* given by the expression $\sigma =a+b\varepsilon_{plastic}^{n}$, with *a*, *b* and *n* representing material parameters acquired by fitting experimental data (see Methods). Indeed, plastic deformation was observed for cases wherein the nucleus was stiff (i.e., *E*_*n*_ ≥ 1 kPa) and the tissue stiffer (i.e., *E*_*T*_ ≥ *E*_*n*_) (Fig 3b). For these cases, dramatic drop in nuclear circularity was observed, i.e., *D*/*L* < 0.6 (Fig 2e). Plastic deformation was also observed for cells with stiff nuclei (*E*_*n*_ = 5 kPa) transiting through moderately stiff matrices (*E*_*n*_ = 1 − 2 kPa); however, localized kinking did not occur for these cases. Plastic nuclear deformation was associated with reduced nuclear stresses (Fig 3c), as well as stresses on the cell membrane (S3 Fig).

Interestingly, profiles of plastically deformed nuclei revealed the presence of kinks at the front edge with large kink formation observed for the cases of *E*_*n*_ ≥ 1 kPa, *E*_*T*_ = (2, 5) kPa (Fig 3b). Interfacial migration (i.e., *E*_1_ ≠ *E*_2_) through stiff matrices was also found to be facilitated by plastic deformation (S4a Fig). A plot of temporal evolution of hoop stress (*σ*_*θθ*_) during pore entry revealed varying stress profiles across the front end of the nuclear membrane marked by the green-black and red-blue dots (Fig 3d). For a stiff nucleus, necking was observed at the lateral edges when it is squeezed to enter the pore (S4b Fig). This was also observed to be the location of initiation of plastic deformation. Necking temporally precedes kink formation at the front edge of the nucleus. For soft nucleus, i.e., *E*_*n*_ = 0.2 kPa, the front end of the nuclear membrane (i.e., green-black dots) was under compressive stresses (negative hoop stress) only. In contrast, for stiff nucleus, i.e., *E*_*n*_ = 2 kPa, while the outer edge of the front end of the nuclear membrane (i.e., blue dot) underwent drastic increase in compressive stresses, the inner edge of the nuclear membrane (i.e., red dot) underwent a sudden switch from compressive to tensile stresses. A bifurcation in stresses (red and blue curves, Fig 3d) in the membrane creates a condition of extreme bending deformations which might be indicative of localized structural disintegration. In addition to highlighting the prominent role of plastic deformation of the nucleus in enabling entry into small pores, our results suggest that buildup of stresses during entry may lead to nuclear membrane damage.

### Nuclear plasticity and DNA damage: insights from experiments

**Fig 4:**
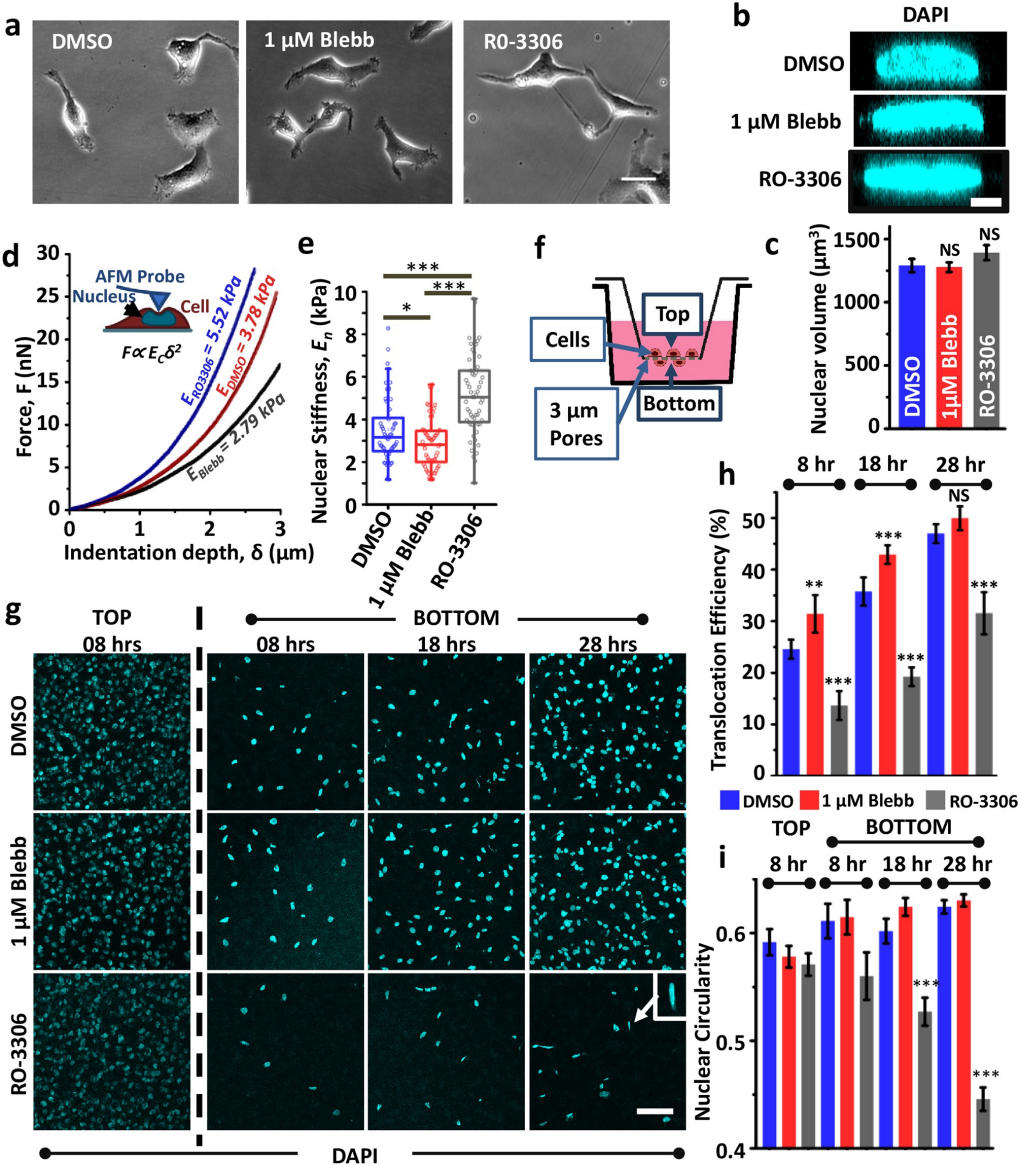
Influence of nuclear stiffness on pore migration efficiency and nuclear plasticity. (a) Phase contrast images of HT-1080 fibrosarcoma cells treated with vehicle (DMSO), 1 *μ*M blebbistatin (Blebb) or 10 *μ*M RO-3306 (RO) for 12 hours. Scale bar = 30 *μ*m. (b) Representative XZ plane images of DAPI stained nuclei of DMSO, Blebb and RO-treated cells. Scale bar = 5 *μ*m. (c) Quantitative analysis of nuclear volume (*n* = 20 − 50 nuclei per condition across 2 independent experiments). Error bars represent ±SEM. Statistical significance was determined by one-way ANOVA/Fisher Test; NS: *p* > 0.05. (d) Probing nuclear stiffness of cells with a stiff pyramidal probe. Cells were treated with DMSO, Blebb or RO for 12 hours prior to experiments. Nuclear stiffness values were estimated by fitting ≥2 *μ*m of indentation data using Hertz model. (e) Quantification of nuclear stiffness of DMSO-treated, Blebb-treated and RO-treated cells (*n* = 40 − 60 nuclei per condition across 2 independent experiments). Error bars represent ±SEM. Statistical significance was determined by one-way ANOVA/Fisher Test; * *p* < 0.05, *** *p* < 0.001. (f) Schematic of transwell migration assay through 3 *μ*m pores; Cells were seeded in the upper chamber containing plain DMEM supplemented with DMSO or drugs. Lower chamber was labelled with DMEM containing 20% serum for creating a chemokine gradient. (g) Representative DAPI stained images of nuclei in upper chamber (referred to as TOP) and lower chamber (referred to as BOTTOM) at 8, 18 and 28 hrs after cell seeding; Scale bar = 100 *μ*m. (h) Quantification of translocation efficiency of DMSO/Blebb/RO-treated cells at 3 different time-points (*n* ≥ 900 nuclei per condition were counted in the upper chamber; experiment was repeated thrice). (i) Quantification of nuclear circularity of DMSO/Blebb/RO-treated cells at the top (8 hr time point) and at the bottom surface of the pores at 3 different time-points (*n* > 80 nuclei per condition; experiment was repeated twice). Error bars represent ±SEM. Statistical significance was determined by one-way ANOVA/Fisher Test; *** *p* < 0.001, ** *p* < 0.01, NS: *p* > 0.05.

To finally compare our simulation predictions with experiments, confined migration experiments were performed using HT-1080 fibrosarcoma cells which are highly invasive and are capable of switching from proteolytic to non-proteolytic migration upon inhibition of protease activity [36]. This switch is enabled by nuclear softening through phosphorylation of Lamin A/C, and can also be induced by treatment with the non-muscle myosin II inhibitor blebbistatin (hereafter Blebb) [6]. To assess the importance of nuclear stiffness and nuclear plasticity during confined migration, experiments were performed in the presence of Blebb and the CDK inhibitor RO-3306 (hereafter RO), which inhibits lamin A/C phosphorylation [37]. Cells treated with DMSO served as controls. At the drug doses used, no obvious differences in cell morphology were observed (Fig 4a). While nuclear volume was preserved across the three conditions (Fig 4b and 4c), AFM probing of nuclear stiffness with a stiff tip right at the center of the cell (above the nucleus), and fitting of ∼ 2 *μ*m of force curves revealed reduction in nuclear stiffness of Blebb-treated cells compared to controls (Fig 4d and 4e). In comparison, RO-treated nuclei were significantly stiffer.

To assess the implications of these alterations in nuclear stiffness on the efficiency of confined migration, transwell migration through 3 *μ*m pores was performed wherein cells were plated on the top of the transwell pores and the fraction of cells reaching the bottom was quantified at three different time-points, i.e., 8, 18 and 28 hours after seeding (Fig 4f and 4g). Cells were stained with DAPI for ease of cell counting as well as for assessing nuclear morphology before and after transit through the pores. Time-snaps of the number of cells that transited through the pores and reached the bottom surface illustrated the clear advantage of nuclear softening during confined migration. While the number of nuclei at the bottom were comparable in DMSO and Blebb-treated cells at all the three time-points, the number of RO-treated nuclei were significantly lesser (Fig 4g). Quantification of translocation efficiency, i.e., the fraction of cells that transited through the pores, revealed Blebb-treated cells to be the most efficient in pore migration, and RO-treated cells to be the least efficient (Fig 4h).

To next assess the possibility of nuclei undergoing plastic deformation during pore migration, nuclei shape was quantified by measuring nuclear circularity as a function of time. Nuclear circularity of DMSO and Blebb-treated cells remained unchanged across the three time-points, and were comparable with cells that remained at the top surface (Fig 4i). Though nuclear circularity of RO-treated cells was comparable to that of DMSO and Blebb-treated cells at the 8 hr time-point, there was a gradual drop in nuclear circularity with time with maximum drop of ≈25% observed at the 28 hour time-point. The dramatic change in nuclear circularity of RO-treated cells suggests that nuclei of these cells have undergone plastic deformation and retain their deformed shapes.

**Fig 5:**
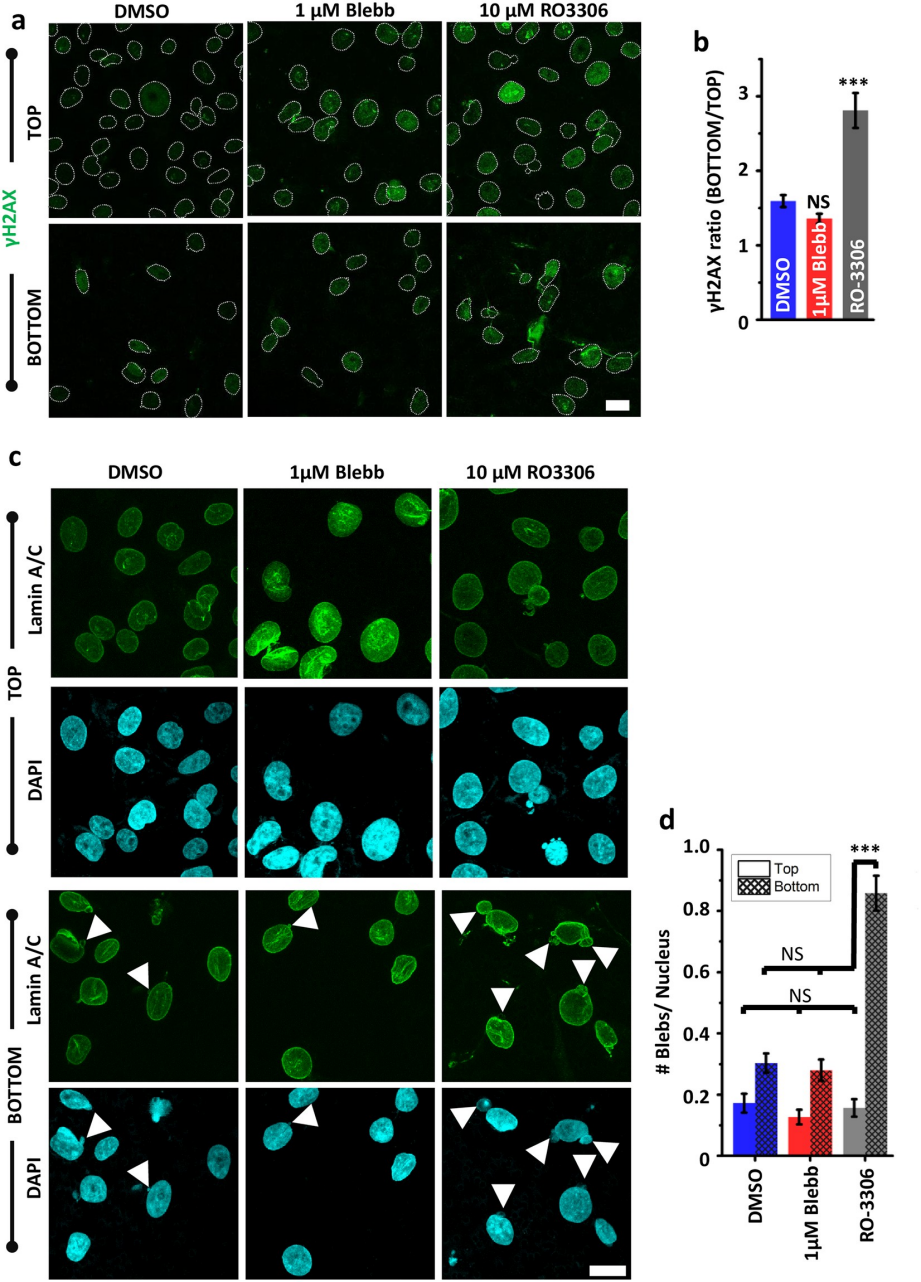
Plastic deformation of the nucleus increases susceptibility to damage. (a) Representative *γ*H2Ax-stained images of DMSO/Blebb/RO-treated cells in upper chamber (referred as TOP) and lower chamber (referred as BOTTOM) of transwell pores 28 hrs after cell seeding. Nuclei are outlined with white dotted lines; Scale Bar = 20 *μ*m. (b) Quantification of ratio of integrated *γ*H2Ax intensity between BOTTOM layer and TOP layer in DMSO/Blebb/RO-treated cells (*n* = 40 − 120 nuclei per condition; experiment was repeated twice). Error bars represent ±SEM. Statistical significance was determined by Mann-Whitney test; *** *p* < 0.001, NS: *p* > 0.05. (c) Representative Lamin A/C (green) and DAPI (blue) stained images of DMSO/Blebb/RO-treated cells in Top and Bottom layer of transwell pores at 28 hrs after cell seeding. White arrows indicate nuclear blebs. Scale bar = 20 *μ*m. (d) Quantification of average number of blebs per nucleus in DMSO/Blebb/RO-treated cells in top and bottom layer of the transwell inserts (*n* > 250 nuclei per condition pooled from two independent experiments). Error bars represent ±SEM. Statistical significance was determined by Mann-Whitney test; *** *p* < 0.001, NS: *p* > 0.05.

To finally probe the link between the nature of nuclear deformation (i.e., elastic versus plastic) and nuclear damage, cells were stained with *γ*H2Ax, a marker of DNA damage, before and after transwell migration (Fig 5a). Quantification of *γ*H2Ax intensity normalized to DMSO condition revealed higher basal level of damage in RO-treated cells, but no change in Blebb-treated cells (S5 Fig). These baseline differences were amplified to different extents after transwell migration. Quantification of the ratio of *γ*H2Ax levels between BOTTOM layer and TOP layer revealed ≈ (30 − 50)% increase in DMSO and Blebb-treated cells (Fig 5b). In comparison, ≈ 200% increase was observed in RO-treated cells. To establish a direct correlation between *γ*H2Ax levels and nuclear damage, nuclei co-stained with Lamin A/C and DAPI were imaged for visualizing formation of nuclear blebs (white triangles, Fig 5c). For all the three conditions, the proportion of nuclei with blebs remained unchanged in cells in the TOP layer, but increased after transwell migration to different extents (Fig 5d). Specifically, the proportion of cells with nuclear blebs increased from ≈ 30% in DMSO/Bleb-treated cells to ≈ 90% in RO-treated cells. Together, these results validate our model predictions and suggest that plastic deformation of the nucleus increases susceptibility to DNA damage.

### Scaling relationships

**Fig 6:**
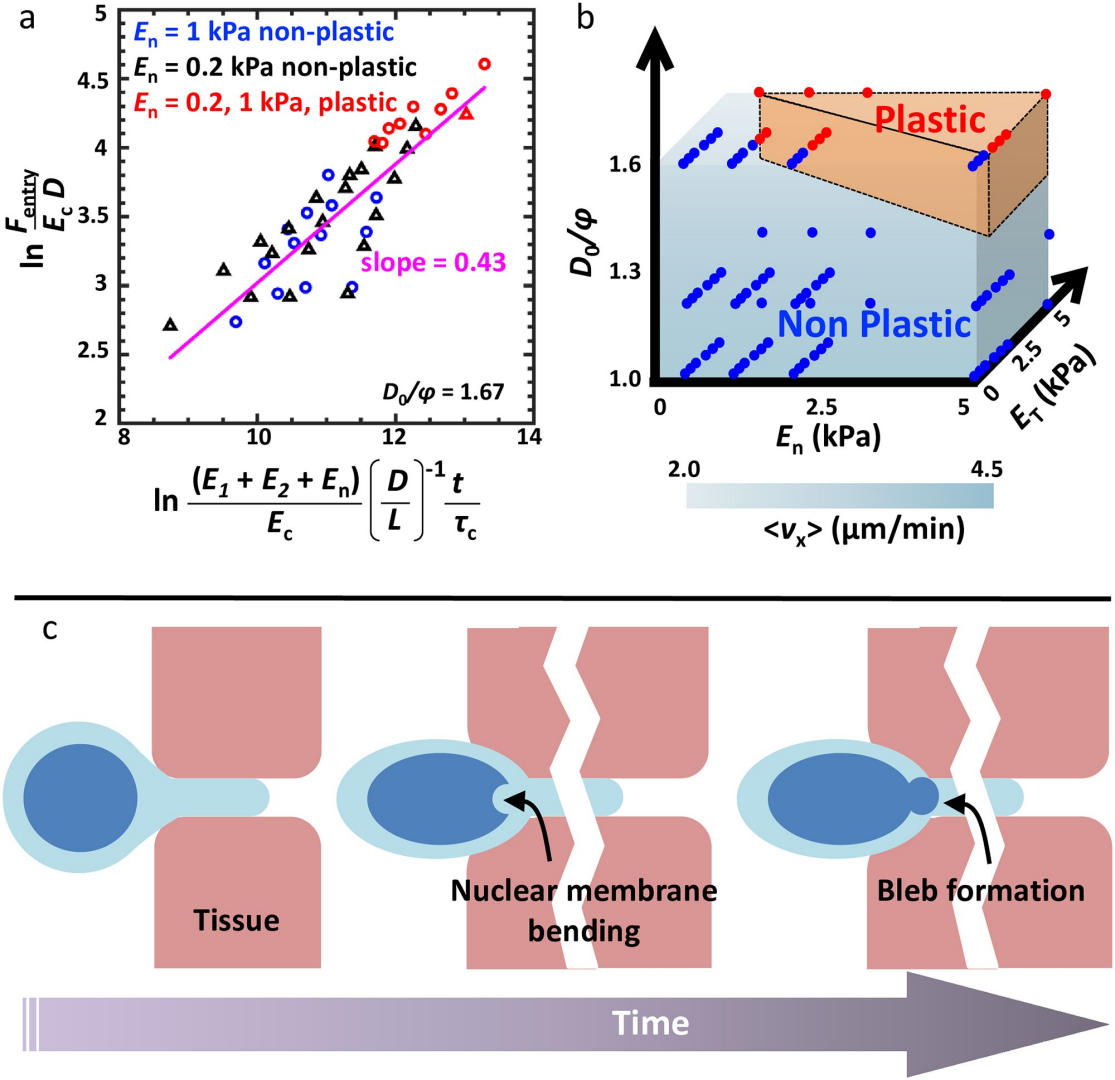
Scaling relationships and proposed model of nuclear damage. (a) Non-dimensional cellular force scaled with possible parameters affecting the cellular force generation during confined migration for *D*_0_/*ϕ* = 1.67. (b) Phase diagram depicting the zones of non-plastic and plastic nuclear deformation required for pore entry for different values of *E*_*n*_, *E*_*T*_ and *D*_0_/*ϕ*. (c) Proposed model of nuclear damage. Compressive forces imposed by the surrounding tissues cause initial nuclear membrane damage. This serves as the precursor to nuclear bleb formation.

The cellular force required for a nucleus to successfully enter a pore is expected to depend on both nuclear stiffness and tissue stiffness. The dynamic change in nuclear circularity over the period of entry into the pore is then a function of the aforementioned factors. The ratio of initial nuclear size to initial pore size (*D*_0_/*ϕ*) is of limited value for analyzing cell migration through deformable matrices because the pore size widens with the passage of a cell nucleus through it. A non-dimensionalized scaling relationship between nuclear circularity (*D*/*L*) and a combination of tissue and nuclear stiffness ($E_{1}E_{2}/E_{n}^{2}$) shows the slopes followed by cells of varying nuclear stiffness (S6a Fig). Force required by a cell of given cell/nuclear stiffness for entering a pore is well fit by the following power law with an exponent of 0.43 (*R*^2^ = 0.75) (Eq 1) (Fig 6a):

$$\begin{array}{c} \frac{F}{E_{c}D}=(\frac{E_{1}+E_{2}+E_{n}}{E_{c}}.\frac{L}{D}.\frac{t}{\tau_{c}}{)}^{0.43} \end{array}$$ (1)

where, *E*_*c*_, *t* and *τ*_*c*_ refer to cytoplasmic stiffness, time of pore entry and viscoelastic time constant of the cytoplasm respectively. The dimensional force itself was found to increase exponentially with *D*/*L* with the exponents dictated by *E*_*n*_ and the extent of plastic deformation (S6b Fig). The magnitude of the exponent was highest for the case of stiff nucleus (*E*_*n*_ = 1 kPa) undergoing plastic deformation, lowest for the case of stiff nucleus undergoing non-plastic deformation, and intermediate for the case of soft nucleus (*E*_*n*_ = 0.2 kPa) undergoing non-plastic deformation. These scaling relationships can be utilized for predicting cell generated forces based on experimentally observed parameters such as nuclear circularity and mechanical properties of various cellular and tissue structures.

## Discussion

The numerical model of cell migration under confinement presented in this study incorporates essential cellular features at the microscale, namely, nuclear elastoplasticity and viscoelasticity of other cellular components and extracellular matrices in addition to stress-stiffening of cytoplasm that make it more realistic than previous FE models (S1 Table). The dramatic increase in instantaneous migration speed of the nucleus observed in our simulations is consistent with experimental observations [38, 39], and can be attributed to the sudden release of the built-up potential energy due to deformation of internal elastic springs in the cell and nucleus and its conversion into kinetic energy. Though the maximum possible intracellular protrusive force (*F*_*P*_) defined in our model is comparable to literature reported values [40, 41], the forces predicted by our model are nearly an order of magnitude lower than the values reported by Lele and co-workers [41]. These differences may arise due to the experimental setup and/or sensitivity of experimental assays. For example, in the aforementioned literature, micropipette aspiration was performed on cells adherent on glass and migrating through stiff PDMS micropillars (*E* ≈ 2 MPa).

Our model predicts that while migrating through matrices stiffer than the nucleus (i.e., *E*_*T*_/*E*_*n*_ > 1), the nucleus undergoes plastic deformation (Fig 6b). Plastic deformation of the nucleus was also observed for the case of a cell with a stiff nucleus (*E*_*n*_ = 5 kPa) migrating through a relatively soft matrix (*E*_*T*_ = 1 − 2 kPa). However, kink formation was observed for the first case only. Long-term change in nuclear circularity of RO-treated cells observed at the 28 hr time-point, but not of DMSO and Blebb-treated cells, is indicative of nuclei of RO-treated cells undergoing plastic deformation. However, at the 8 hr time-point, nuclear circularity of RO-treated cells which transited to the bottom of the pores was comparable to that of DMSO and Blebb-treated cells as well as with that of cells at the top. Although RO-3306 inhibits CDK1 and perhaps other CDKs to affect cell cycle as well as nuclear stiffness with the observed result of increased DNA damage (Bottom/Top) (Fig 5b), inhibition of cell cycle with a different drug that does not target CDK1 was found to have no effect on DNA damage [22]. Given the broad heterogeneity in nuclear stiffness measurements, we speculate that RO-treated cells which reached the bottom of the transwell pores at the 8 hr time-point correspond to a sub-population of cells with softer nuclei which underwent elastic deformation during pore migration.

Since Lamin A/C levels scale with tissue stiffness [42], our results of cells with stiff nuclei migrating through stiffer tissues correspond to cancers such as osteosarcoma, wherein migration-induced DNA damage has been shown to cause genomic heterogeneity [43]. Stiff nuclei have been reported to result from increased lamin A concentration in the nucleus [20], especially in the genetic mutations caused in the Hutchinson Gilford Progeria Syndrome (HGPS) [44, 45]. In comparison, the absence of nuclear kinks in cells with soft nuclei passing through stiff matrices suggests that nuclear softening may represent a robust strategy utilized by cells to migrate through pores without undergoing nuclear membrane rupture. Consistent with this idea, *γ*H2Ax levels and the average number of nuclear blebs were comparable in control and Blebb-treated cells, but significantly elevated in RO-treated cells.

The relative insensitivity of average cell speed to *E*_*n*_/*E*_*T*_ suggests that tissue stiffness-dependent temporal tuning of nuclear stiffness by lamin A/C phosphorylation may enable cancer cells to migrate at comparable efficiency through tissues of varying composition and pore sizes. Approximating the nuclear membrane and the lamina as a simply supported elastic plate of thickness *h*, the flexural rigidity (*F*_*D*_) can be given as [46]:

$$\begin{array}{c} F_{D}\approx \frac{Eh^{3}}{\left(1-\nu^{2}\right)} \end{array}$$ (2)

where, *E* and *ν* are elastic properties of the nuclear lamina. Since thickness *h* has a relatively greater influence on flexural rigidity compared to stiffness *E*, a thin lamina is more prone to damage due to bending than a soft lamina. This might explain increased blebbing due to nuclear damage reported in laminopathies (loss of lamin A/C) but not in immune cells or cancers with soft nuclei.

The chromatin contained within the nucleus is a major determinant of nuclear deformation. Mechanotransduction between the actin cytoskeleton and nucleoplasm through the interconnecting LINC complex has been shown to play a critical role in chromatin dynamics during DNA repair [47] and gene transcription [48, 49]. The spatial organization of the chromatin changes with nuclear stress and shape change. Compact chromatin network acts as an elastic spring to resist small deformations [49]. While small strains lead to strain stiffening of nuclei due to chromatin compaction [50], large deformation of nuclei is facilitated by the actin and vimentin cytoskeleton [51] and Lamin A/C [50]. Change in spatial organization of chromatin fibers can possibly lead to permanent plastic deformation of nuclei. A full rupture in the membrane allows the intranuclear pressure to become more than the intracellular pressure, thus facilitating the leakage of genetic material into the cytosol. An alternate mechanism is also observed in cells migrating under confinement where the nuclear membrane gets mechanically decoupled from the nuclear lamina which leads to membrane blebbing due to chromatin flow into this vacant pocket of space [52]. This experimental observation can be linked to our model prediction where we find that the nuclear lamina bends when the nucleus undergoes plastic deformation. This extreme nuclear bending as seen in Fig 3b and 3d might result in delamination of the nuclear cortex from the nuclear membrane leading to bleb formation.

Nuclear blebbing has also been reported to be caused by influx of water into the nucleus under confinement [53]. In this study, we did not consider the effect of water influx into the nucleus, the characteristic time (*τ*) for which is found to be governed by the equation: $\tau =r_{n}^{2}/D_{c}$, where *r*_*n*_ is the radius of nucleus and *D*_*c*_ is the diffusion coefficient of water. Thus, for an undeformed nucleus of *r*_*n*_ = 2.5 *μ*m and *D*_*c*_ = 50 *μ*m^2^/s [54], *τ* = 0.125 *s*, which is extremely small compared to the timescales of migration (minutes to hours), even smaller for deformed nuclei. Moreover, since we consider a quasi-static viscoelastic description of the model, we did not consider the transient poroelasticity of nuclei or cytoplasm. Our results show that there is a time-dependent spatial gradient of compressive forces on the nuclear lamina due to actomyosin fibres during nuclear entry into a pore. In such a situation, by the time the nucleus completely enters into the pore, the front of the nucleus relative to the direction of migration has been under compressive stresses much longer than the rear. Therefore, the probability of nuclear membrane rupture at the nuclear front is much higher than at the rear as has been consistently reported in experiments. Compressive stresses beyond a critical threshold (yield stress *σ*_*y*_) causes the nucleus to yield and this yield zone is also found to spread starting from the frontolateral region of the nucleus where necking occurs due to constriction to the rear of the nucleus (S4b Fig). In case of stiff nuclei transiting through stiffer matrices (*E*_*n*_ ≥ 1 kPa, *E*_*T*_ ≥ *E*_*n*_), plasticity-induced deformation of the nuclear membrane leads to buckling and finally membrane failure. The combination of nuclear stress profiles and *γ*H2Ax results suggests that plastic nuclear deformation that initiates at the nuclear lamina gets propagated throughout the nucleus.

Nuclear membrane rupture in micropipette aspiration experiments has been attributed to tensile stresses at the anterior periphery of the nucleus [23, 55]. Similar results have been recapitulated by Cao et. al. [15] in their model where they proposed that the front and lateral edges of the nucleus might be susceptible to tensile-stress induced damage during migration through ECM-like environments. Though these findings implicate tensile stresses as a factor contributing to nuclear damage, nuclei are primarily subjected to compressive forces during confined migration. Our observations of kink formation at the tip of the nuclear membrane proximal to the direction of migration correlates with experimental observations of the spatial location of nuclear damage during migration through extreme confinement in stiff environments [18, 19, 56]. Since kink formation is expected to occur between the stages of nuclear compression, subsequent blebbing and eventual rupture, resolving it temporally during experiments is challenging. However, there might be some indication of kink formation in-vitro in a study by Lammerding and co-workers [21] where they show that lamin B is depleted from the region where membrane bleb is formed. They suggest that this is due to detachment of lamina from the membrane. In our simulations, this detachment can be related to mechanical delamination (similar to delamination in composite structures) under compression. The kink formation might be a consequence of excessive bending of the nuclear lamina driven by the combined effects of tissue stiffness and the peri-nuclear cytoskeleton. Formation of smaller lateral kinks might aid in the initiation of plastic deformation. We propose that rapid buildup of compressive and tensile stresses at the point of pore entry induces nuclear envelope damage; subsequent localized delamination of the lamina from the nuclear membrane may serve as a precursor to experimentally observed nuclear blebbing (Fig 5c). The genetic material, already under significant external pressure and previously held back by the structural integrity of the nuclear lamina, then oozes out through the damaged orifice to form a bleb that may eventually rupture subject to membrane tension. However, unless the damage is extreme, nuclear rupture is repaired using ESCRT machinery [18, 21, 52]. Our results suggest that compressive stress-induced membrane damage and nuclear blebbing only occurs in a specific window depending on nuclear/tissue stiffness and extent of confinement, and may be critical for migration through stiff environments. Our experimental observations indeed support this idea as change in nuclear circularity indicative of plastic deformation of the nucleus was only observed in RO-treated cells, where DNA damage was maximum. The lack of plastic deformation in Bleb-treated cells which were more invasive and had lesser DNA damage suggests that nuclear softening may be a more effective invasion strategy compared to nuclear plasticity.

In conclusion, we have developed a numerical model of confined cell migration that contributes to our understanding of the underlying physics of nuclear deformation and stresses during confined migration. We further validate our key prediction of nuclear plasticity leading to nuclear damage using experiments wherein RO-induced nuclear stiffening led to plastic deformation and higher DNA damage. Our model suggests that nuclear membrane damage in stiff nuclei plastically deformed by compressive stresses, may serve as the precursor for bleb formation that ultimately facilitates successful migration of a cell through stiff tissues.

## Materials and methods

### Computational methods

For studying dynamics of confined cell migration, a plane strain finite element (FE) model of the system was created in ABAQUS/Explicit. FE models involve discretizing the system into smaller elements by meshing it (dividing the system into several discrete polygonal elements/parts). Numerical techniques (e.g., Runge-Kutta technique) are then used to arrive at an approximate solution to an equation of the general form [*K*]{*u*} = {*F*}. This is analogous to a Hookean spring with [*K*] being a matrix representing spring stiffness, {*u*} representing a displacement vector and {*F*} denoting a vector of applied force. This equation is computed at each node of each polygonal element that the object is made of (S1 Text). Discontinuities resulting due to cellular organelles and granular structures at the nanoscale are homogenized and considered as a continuum at the microscale.

A computational domain needs to be selected for numerical simulations in FEM so that boundary conditions (BCs) are applied to the PDEs that are solved as part of the problem. Since our desired direction of cell migration is the + *x*–direction, we assume that the deformation or volume change of the cell perpendicular to the plane of migration (*xy*–plane) would be much lower than that in plane and hence can be neglected. This is complemented by our chosen material parameters of all system components where the Poisson’s ratio (*ν*) is 0.3 indicating that all the materials are compressible (S2 Table), that is, a change in area in the *xy*–plane does not accompany a similar change in the *yz*− or *xz*–planes. This assumption reduces the complexity of the system from 3D to a 2D plane strain problem. This assumption also finds credibility in experimental observations of cell migration through microchannels where the cell deforms or gets polarized in the direction of migration but the accompanying lateral deformation is negligible [57, 58].

In our formulation, entry of a 10 *μ*m diameter cell with a 5 or 6 *μ*m diameter nucleus into a pore ({3, 5} *μ*m diameter) at the interface of two tissues was simulated with the system assumed to be in a quasi-static state for the entire duration of the simulation (S1 Fig). This was verified by observing the kinetic energy of the entire system to be much lower than its internal energy. Pore entry was mediated by active protrusive forces generated by the cell at the cell front [59, 60]. A comparison of the salient features of two other FE models [15, 61] with our model is presented in S1 Table.

The cell is composed of the following components: cell membrane, cytoplasm, nuclear membrane and nucleus. All the components except the nucleus are approximated as Kelvin-Voigt viscoelastic elements [62] where stress developed in a system depends on the strain and strain rate and is given by the equation $\sigma_{ij}=K\varepsilon_{ij}+\eta {\dot{\varepsilon }}_{ij}$. Here, *K* is an elasticity modulus that corresponds to spring or solid stiffness and *η* is viscosity of the constituent fluid. The subscript “*ij*” refers to the Einstein convention in the spatial dimension. The viscoelastic character of each component in the system is represented in the form of normalized creep compliance (S1c Fig). Creep is a characteristic feature of a viscoelastic material that defines the amount by which a system deforms under persistent stress. Compliance is the reciprocal of stiffness (Pa) and is a measure of the ease with which a body deforms under stress.

The nucleus is considered to be elastoplastic with its behaviour described by a strain hardening power law equation $\sigma =a+b\varepsilon_{plastic}^{n}$, similar to Ludwik’s equation [63]. The plastic strain is denoted by *ε*_*plastic*_. The coefficients *a*, *b* and the exponent *n* were estimated to be equal to 41 Pa, 17 Pa and 2.89 respectively (*R*^2^ = 0.9981), with data trend similar to those reported by [29]. The total strain in the system is the sum of both elastic and plastic strains (*ε*_*total*_ = *ε*_*elastic*_ + *ε*_*plastic*_). Yield stress (*σ*_*yield*_) is defined as the stress at which a substance develops permanent plastic deformation. When there is no plastic strain in the nucleus (i.e., nucleus deforms elastically), *σ* = *A* = *σ*_*yield*_. When *σ* < *σ*_*yield*_, then *σ* = *Eε*_*elastic*_, where, *ε*_*elastic*_ < *σ*_*yield*_/*E*. Plastic strain *ε*_*plastic*_ is given by: *ε*_*plastic*_ ≥ *σ*_*yield*_/*E*. In a strain hardening material, the yield stress increases with strain, thus implying that it becomes progressively difficult to strain the material.

The elastic properties of the nucleus arise from the lamin network below the nuclear membrane along with chromatin fibres. Plastic nature of nuclei have been reported in several studies demonstrating the irreversible change in shape of nuclei deformed under stress [29, 30, 50]. A recent study [50] also demonstrated that nuclei stiffen progressively with strain, a phenomenon attributed to chromatin compaction at small strains and to Lamin A/C at large strains. Plastic deformation in non-fibrous biological materials arise due to irreversible dislocation or dislodgement of molecules from their unperturbed positions. In fibrous biological materials like collagen, plasticity under tensile strains is caused due to unentanglement of fibers [12]. The tissues representing the two sides of the interface are also modelled to be viscoelastic. A Poisson’s ratio of *ν* = 0.3 which is a typical value considered for compressible biomaterials [15], was chosen for the cellular components as well as the two tissues. This value also sits well with our assumption that the out-of-plane volume change is negligible compared to the in-plane deformation. All elastic material properties are listed in S2 Table.

Cells are known to exert forces by actomyosin contraction resulting from myosin motors sliding on actin filaments [10, 11]. During migration, cells regulate F-actin polymerization and generate protrusions [64], leading to an increase in force generated. Crosslinking of actin filaments with proteins such as *α*-actinin, filamin and scruin stiffens these fibers and they bundle together to generate protrusive forces. Myosin II plays an important role in creation and regulation of stress fibers and force generation [64, 65]. To mimic these phenomena in our model, we assumed that the cell generates force (*F*_*P*_) at the leading edge in the direction of motion (x-axis) in a smooth monotonic fashion (S1d Fig). *F*_*P*_ was assumed to be generated in a distributed fashion at the cell front as shown in S1d Fig such that the magnitude of the maximum force (≈ 6.4 nN) generated by the cell remained in the physiologically relevant range [40, 41]. Cytoskeletal stress stiffening was implemented in our model using the ABAQUS/Explicit subroutine VUSDFLD (S1 Text, S1 Fig). In each time increment, the model algorithm checks if the cytoplasmic shear stress increased beyond 20 kPa, the cytoplasmic shear stiffness was increased in discrete steps from 1.0001 Pa to 1.1 Pa, and the system re-equilibrated (S1e and S3 Figs) [31, 32, 66]. This variation can be curve-fit as per the equation *log*_10_(*E* − 1) = 49.41 *log*_10_ *σ*_shear_ − 68.31, where *E* and *σ*_*shear*_ represent cytoplasmic stiffness and shear stress, respectively. The simulation was stopped once the nucleus enters the pore completely.

An explicit formulation was implemented to successfully resolve large nonlinear deformations in meshes in the Lagrangian or material domain. In this energy-based formulation, the stable time increment (Δ*t*) to solve the numerical problem depends on the stress wave velocity through the smallest element in the mesh (Δ*t* ≈ *L*_*min*_/*c*_*d*_), where *L*_*min*_ is the smallest element dimension in the mesh and *c*_*d*_ is the dilatational wave speed through the element. Mass scaling was used to ensure that Δ*t* was of the order O(-4). Frictionless hard contact was assumed at the cell-gel interface to simulate non-adherence of cell to the gel or channel walls. The cell surface was thus allowed to separate after contact with the gel surface. For the cases of *D*_0_/*ϕ* = 1 and 1.67, a total of 31183 bilinear plane strain CPE4R elements were used in the model, of which the two tissues were composed of 7469 and 7366 elements and the cell was composed of 16348 elements. For the case of *D*_0_/*ϕ* = 1.2, while the two tissues had the same number of elements as mentioned above, the cell was composed of 15870 elements, leading to a total of 30705 CPE4R elements in the model. A mesh sensitivity analysis was done on the nuclear membrane and the cell membrane to arrive at the optimal mesh element dimensions to minimize mesh distortion. The optimal minimum element dimension was found to be 0.005 *μ*m and the maximum was 20 *μ*m. The mesh size was modulated so as to be fine in the regions that were expected to come in contact or that would undergo large deformation. The nuclear membrane as well as the cell membrane, 0.05 *μ*m in thickness, had 10 elements in the through-thickness direction to mitigate the effects of excess artificial bending stiffness of the membranes. This value for the optimum number of elements was arrived at using mesh sensitivity analysis. Additionally, a distortion control algorithm in-built in ABAQUS was used to counter mesh distortions (prevent element inversion and excessive distortion) when minimum to maximum dimension ratio of a mesh element decreased below 0.1. This was done to ensure that the kink in the nuclear membrane observed in our results is not a numerical artifact of the FEM simulation.

### Experimental methods

#### Cell culture and reagents

HT-1080 fibrosarcoma cells obtained from National Center for Cell Science (NCCS) (Pune, India), were cultured in DMEM (high glucose, Invitrogen) containing 10% FBS (Hi-media). For nuclear stiffness experiments, cells were plated sparsely on glass coverslips coated with rat-tail collagen I (Cat # 3867, Sigma) at a coating density of 10 *μ*g/*cm*^2^. Cells were incubated with DMSO (i.e., vehicle), 1 *μ*M blebbistatin (Cat # B0560, Sigma) or 10 *μ*M RO-3306 (Cat # ab141491, Abcam) for 12 hours prior to probing with AFM.

#### Atomic Force Microscopy (AFM) and imaging

For measuring nuclear stiffness, stiff tips (32 kHz, TR400PB, Asylum Research) with nominal stiffness of 120 pN/nm were used, with exact values of cantilever stiffness determined using thermal calibration method. Cells were indented towards the center right on top of the nucleus, and indentation data more than 2000 nm were fitted with Hertz model to obtain estimates of nuclear stiffness.

For transwell migration studies, 10^5^ cells were seeded on the upper chamber of 24 well plate cell culture inserts containing 3 *μ*m pores (Cat # 353096, Merck). The inserts were coated with rat-tail collagen I. For creating a gradient, the upper chambers were filled with plain DMEM supplemented with drugs and the lower chambers filled with DMEM containing 20% FBS. After 8, 18 and 28 hrs, cells were fixed with 4% PFA and then stained with DAPI for 45 minutes. After washing with PBS, membrane was cut and mounted on a glass slide using mounting media. Confocal z-stack images were acquired at 20x magnification using Scanning Probe Confocal Microscope (Zeiss, LSM 780) at identical exposure and gain settings. Images analysis and quantification was performed using Fiji-Image J software. Translocation efficiency was calculated using the equation $\frac{N_{b}}{N_{t}+N_{b}}\times 100$ where *N*_*t*_ and *N*_*b*_ represent the number of DAPI-stained nuclei on the top and bottom surfaces of the membrane per frame.

For *γ*H2Ax and Lamin A/C staining, fixed cells were permeabilized with 0.1% Triton-X 100 for 8-10 mins, blocked with 2% bovine serum albumin (BSA) for 1 hr at room temperature, and then incubated with *γ*H2Ax rabbit monoclonal antibody (Cat # 9718S, CST) and anti-Lamin A/C mouse monoclonal antibody (Abcam, Cat # ab8984) overnight at 4°C. The following day, after washing with PBS, Alexa-Fluor 488 anti-rabbit IgG and Anti-Mouse Alexa fluor 555 was added for 2 hr at room temperature. Nuclei were stained with DAPI for 5 min at room temperature. Images were acquired at 40x magnification using Scanning Probe Confocal Microscope (Zeiss, LSM 780) at identical exposure and gain setting.

## Supporting information

S1 Text Supporting text. (PDF)

S1 Table Supporting table of comparison with other FE-based cell migration models. (PDF)

S2 Table Supporting table of material parameters. (PDF)

S1 Fig Model definition. (a) Dimensions of various parts of the modelled cell. (b) Finite element model with mesh. Lateral and transverse boundaries of the tissue (1 and 2) are constrained in their perpendicular directions. (c) Viscoelastic properties of various materials in the model. (d) Temporal variation of input force. (e) Simulation process flow. (f) Assumed dependence of cytoplasmic stiffness (*E*_*c*_) with shear stress (*σ*_shear_) encountered by the cell. *E*_*c*_ is increased in discrete steps as indicated by datapoints and a smooth curve is interpolated, i.e., the points are used to define a function between the two variables.
(TIF)

S2 Fig Hoop stresses developed in the cell membrane just after pore entry. For a confinement of *D*_0_/*ϕ* = 1.67, the spatial variation of hoop stresses along the length of the membrane for different combinations of *E*_*T*_ and *E*_*n*_.
(TIF)

S3 Fig Stresses developed in the cytoplasm and the nucleus during pore entry. The spatiotemporal evolution of stress distribution during and just after entry of the 5*μ*m nucleus into a 3*μ*m pore, i.e., *D*_0_/*ϕ* = 1.67. Contours and colourbars indicate von Mises stresses (*σ*_*Mises*_) developed in the cytoplasm and nucleus. The cell membrane has not been displayed in the figures for clarity.
(TIF)

S4 Fig Plastic deformation of nuclei in cells migrating through an interface and spatiotemporal evolution of plastic deformation. (a) *E*_1_ and *E*_2_ refer to the Young’s moduli of tissues 1 and 2 on both sides of the interface. *D*_0_/*ϕ* = 1.67 for all the cases. Contours represent the spatial distribution of plastic strain (*ε*_*plastic*_). (b) Plastic strain accumulated in a cell as a function of time during constricted migration for *D*_0_/*ϕ* = 1.67. *E*_*n*_ = *E*_*T*_ = 2 kPa. Red arrows indicate the region where necking first occurs and plasticity is initiated. The colourbar indicates magnitude of plastic strain in the nucleus (*ε*_*plastic*_ = *ε*_*total*_ − *ε*_*elastic*_).
(TIF)

S5 Fig Quantification of *γ*H2AX expression intensity normalized to DMSO condition at the TOP layer. (TIF)

S6 Fig Scaling relationship of nuclear circularity. Scaling between nuclear circularity and (a) the coupled effect of tissue and nuclear stiffness, and (b) force required by a cell to enter a pore. All datapoints refer to the condition *D*_0_/*ϕ* = 1.67. *E*_1_ and *E*_2_ vary from 0.13 to 5 kPa.
(TIF)

S1 Data Excel spreadsheet containing, in separate sheets, the underlying numerical data and statistical analysis for Figure panels 1c, 1d, 1f, 1g, 2a, 2b, 2d, 2e, 3c, 3d, 4c, 4d, 4e, 4h, 4i, 5b, 5d, 6a, S1c, S1f, S2, S5, S6a, and S6b. (XLSX)
