# Supplementary material for: Nuclear plasticity increases susceptibility to damage during confined migration
Source: PLoS Comput Biol. 2020 Oct 9;16(10):e1008300. doi: 10.1371/journal.pcbi.1008300 (PMC7577492; doi:10.1371/journal.pcbi.1008300)
Supplement: S1 Text — (PDF) [file pcbi.1008300.s001.pdf]

Supplementary Information for Nuclear Plasticity Increases Susceptibility to Damage During Confined Migration

Abhishek Mukherjee[1,2,3], Amlan Barai[4], Ramesh K Singh[2], Wenyi Yan[3*], Shamik Sen[4**]

The Finite Element Method

The Finite Element Method (FEM) is a numerical technique that discretizes an object or structure with complex geometry into several small parts over which partial differential equations (PDEs) that pertain to the specific physics problem can be solved. A PDE (also referred to as strong form) describing the physical problem is converted to an integral of a lower differentiation order (weak form) which is then discretized into small elements. Thus, the integral or weak form gives way to a summation of the parts or elements to eventually result in a general set of simultaneous algebraic equation of the form:

$$[K]\{u\} = \{F\} \; or, \{u\} = [K]^{-1}\{F\} \tag{1}$$

Eq 1 is analogous to a Hookean spring with $[K]$ being a matrix representing spring stiffness, $\{u\}$ representing a displacement vector and $\{F\}$ denoting a vector of applied force although it is general and can pertain to several physical systems. This equation is computed at each node of each polygonal element that the object is made of. All material constitutive models are converted into the form of Eq 1 that guides the relation between stress and strain in the system.

Viscoelasticity of cell and extracellular matrix (ECM)

Cell membrane (plasma membrane) and nuclear membrane are lipid bilayers that are dotted with various protein complexes and ion channels that allow for the transmigration of molecules. In a coarse-grained scenario, the cell membrane can be considered to be composed of a combination of a lipid bilayer, glycocalyx (polymer chains of glycolipids and glycoproteins [1]) and the actin cytoskeleton meshwork attached to the lipid bilayer. This composite cell membrane behaves as a viscoelastic material that flows like a viscous liquid over a short time duration but exhibits a solid-like elastic behaviour at sufficiently long timescales. This leads to the experimental observation of cells attaching themselves onto 2D substrates forming stable shapes [2, 3, 4]. A similar argument can be extended for the choice of the nuclear membrane and nuclear lamina composite as a viscoelastic material due to the similarities in their intrinsic composition with cytoskeleton and cell membrane.

Considering the cell as a closed system consisting of a fibrous mixture (actin cytoskeleton, actomyosin fibres, microtubules and intermediate filaments) and a solvent (cytosol) with no net transport of molecules through the cell membrane, we model it as viscoelastic solid as opposed to poroelastic that assumes a net flux of solvent molecules. The tissue(s) through which the cell migrates is/are also considered as viscoelastic solids because we consider them to be individually closed systems which if stressed, lead to solvent molecules in the vicinity of the stressed region to get displaced from their initial locations temporarily before returning to their original position after stress is relieved. These assumptions are consistent with several experimental studies have demonstrated the viscoelastic nature of cells and tissues.

Viscoelasticity formulation in the time-domain

To describe the constitutive relationship governing an isotropic viscoelastic material, we define the deviatoric and volumetric parts of the stress tensor. For the time-dependent deviatoric stress, time-varying shear strain $\gamma_{dev}(t)$ and shear stress $\sigma_{dev}(t)$ are related as:

$$\sigma_{dev}(t) = \mathrm{G}_0 \int_0^t \mathrm{g}_\mathrm{R}(t-s)\dot{\gamma}_{dev}(s)\mathrm{d}s \tag{2}$$

where G_0 is the instantaneous shear modulus and $g_R(t) = G_R(t)/G_0$ is the dimensionless time-dependent shear relaxation modulus of the viscoelastic material. The time-dependent volumetric behaviour (σ_{vol}) of the material is defined as a change in hydrostatic pressure ($p(t)$) over time and is given by the equation:

$$\sigma_{vol}(t) = p(t) = -\mathrm{K}_0 \int_0^t \mathrm{k}_\mathrm{R}(t-s)\dot{\epsilon}_{vol}(s)\mathrm{d}s \tag{3}$$

where K_0 is the instantaneous bulk modulus and $k_R(t) = K_R(t)/K_0$ is the dimensionless time-dependent bulk relaxation modulus of the viscoelastic material. The instantaneous moduli G_0 and K_0 are related to the Young's modulus E_0 and Poisson's ratio ν as $G_0 = E_0/2(1+\nu)$ and $K_0 = E_0/3(1-2\nu)$ respectively. A viscoelastic material is defined by a Prony series expansion of the dimensionless relaxation modulus given by the equation:

$$g_R(t) = 1 - \sum_{i=1}^{N} g_i^P (1 - \exp^{-t/\tau_i^G}) \tag{4}$$

where N, g_i^P and τ_i^G, $i = 1, 2, ..., N$, are material constants. The shear stress then is given by:

$$\sigma_{dev}(t) = \mathrm{G}_0 \left(\gamma_{dev} - \sum_{i=1}^{N} \gamma_i \right) \tag{5}$$

where $\gamma_i = \frac{g_i^P}{\tau_i^G} \int_0^t \exp^{-s/\tau_i^G} \gamma_{dev}(t-s)\mathrm{d}s$. A similar expression can be acquired for the volumetric response, as shown below:

$$\sigma_{vol}(t) = p(t) = -\mathrm{K}_0 \left(\epsilon_{vol} - \sum_{i=1}^{N} \epsilon_i \right) \tag{6}$$

where, $\epsilon_i = \frac{k_i^P}{\tau_i^K} \int_0^t \exp^{-s/\tau_i^K} \epsilon_{vol}(t-s)\mathrm{d}s$.

Plasticity of the nucleus

Previous studies have demonstrated that stressed nuclei undergo plastic deformation, i.e., they are irreversibly deformed under the application of stresses [5, 6]. Plastic deformation in non-fibrous biological materials arise due to irreversible dislocation or dislodgement of molecules from their unperturbed positions. In fibrous biological materials like collagen, plasticity under tensile strains is caused due to un-entanglement of fibers [7]. Plasticity is generally quantified as a strain or stress regime that extends beyond a critical threshold elastic limit below which molecular dislocations are reversible. An elastic material is assumed to have a linear stress-strain curve within a threshold termed as the proportional limit, beyond which the slope of the curve changes and the relation may become nonlinear. Plastic deformation leads to energy dissipation and therefore, the onset of plasticity signifies a new stable energy state for the material from the previous metastable strained state. Plasticity induced nuclear damage and rupture due to extreme stresses originating under confinement, for instance, may lead to genetic perturbation [8, 9].

Cytoskeletal strain stiffening

Actin bundling proteins (ABPs) get attached to actin filaments with increasing stresses in the cytoplasm [10, 11]. Moreover, actin filaments frequently bundle together in a direction perpendicular to the direction of application of external force. These mechanisms contribute to the eventual stiffening of actomyosin networks. Studies indicate that depending on the actin concentration and crosslinking density the stiffness of such crosslinked fibres can change drastically [10, 11, 12]. In our model, using the ABAQUS/Explicit subroutine VUSDFLD, we implemented this experimental observation such that if the cytoplasmic shear stress increased beyond 20 kPa, the cytoplasmic shear stiffness was increased in discrete steps from 1.0001 Pa to 1.1 Pa, and the system re-equilibrated as shown in S1e Fig.

A comparison of the salient features of two other models [13, 14] besides our model is presented in S1 Table, where 'Y' signifies the feature being accounted for in that model while 'N' implies absence of that feature. While this list is not exhaustive, it lists some of the mechanically and physically critical features that aid in cell migration in 3D matrices.

References

[1] Shurer CR, Kuo JCH, Roberts LM, Gandhi JG, Colville MJ, Enoki TA, et al. Physical Principles of Membrane Shape Regulation by the Glycocalyx. Cell. 2019;177(7):1757–1770.

[2] Desprat N, Richert A, Simeon J, Asnacios A. Creep function of a single living cell. Biophys J. 2005;88(3):2224–2233.

[3] Hoffman BD, Massiera G, Van Citters KM, Crocker JC. The consensus mechanics of cultured mammalian cells. Proc Natl Acad Sci U.S.A. 2006;103(27):10259–10264.

[4] Kasza KE, Rowat AC, Liu J, Angelini TE, Brangwynne CP, Koenderink GH, et al. The cell as a material. Curr Opin Cell Biol. 2007;19(1):101–107.

[5] Pajerowski JD, Dahl KN, Zhong FL, Sammak PJ, Discher DE. Physical plasticity of the nucleus in stem cell differentiation. Proc Natl Acad Sci U.S.A. 2007;104(40):15619–15624.

[6] Tocco VJ, Li Y, Christopher KG, Matthews JH, Aggarwal V, Paschall L, et al. The nucleus is irreversibly shaped by motion of cell boundaries in cancer and non-cancer cells. J Cell Physiol. 2018;233(2):1446–1454.

[7] Kim J, Feng J, Jones CA, Mao X, Sander LM, Levine H, et al. Stress-induced plasticity of dynamic collagen networks. Nat Commun. 2017;8(1):1–7.

[8] Raab M, Gentili M, de Belly H, Thiam HR, Vargas P, Jimenez AJ, et al. ESCRT III repairs nuclear envelope ruptures during cell migration to limit DNA damage and cell death. Science. 2016;352(6283):359–362.

[9] Xia Y, Pfeifer CR, Zhu K, Irianto J, Liu D, Pannell K, et al. Rescue of DNA damage after constricted migration reveals a mechano-regulated threshold for cell cycle. J Cell Biol. 2019;218(8):2545–2563.

[10] Gardel M, Shin JH, MacKintosh F, Mahadevan L, Matsudaira P, Weitz D. Elastic behavior of cross-linked and bundled actin networks. Science. 2004;304(5675):1301–1305.

[11] Gardel M, Shin JH, MacKintosh F, Mahadevan L, Matsudaira P, Weitz D. Scaling of F-actin network rheology to probe single filament elasticity and dynamics. Phys Rev Lett. 2004;93(18):188102.

[12] Gardel ML, Kasza KE, Brangwynne CP, Liu J, Weitz DA. Mechanical response of cytoskeletal networks. Methods Cell Biol. 2008;89:487–519.

[13] Cao X, Moeendarbary E, Isermann P, Davidson PM, Wang X, Chen MB, et al. A chemomechanical model for nuclear morphology and stresses during cell transendothelial migration. Biophys J. 2016;111(7):1541–1552.

[14] Zhu J, Mogilner A. Comparison of cell migration mechanical strategies in three-dimensional matrices: a computational study. Interface Focus. 2016;6(5):20160040.
