# Supplementary material for: Nuclear plasticity increases susceptibility to damage during confined migration
Source: PLoS Comput Biol. 2020 Oct 9;16(10):e1008300. doi: 10.1371/journal.pcbi.1008300 (PMC7577492; doi:10.1371/journal.pcbi.1008300)
Supplement: S1 Table — (PDF) [file pcbi.1008300.s002.pdf]

S1 Table. Comparison with other FE-based cell migration models

Model Feature	Zhu and Mogilner [1]	Cao et. al. [2]	Current Study
Consideration of cytoskeleton	Y	N	Y
Cytoskeletal stiffening	N	N	Y
Nuclear Plasticity	N	Y	Y
Viscoelastic cellular components	N	N	Y
Viscoelastic gel/tissue/ECM	N	N	Y

References

[1] Zhu J, Mogilner A. Comparison of cell migration mechanical strategies in three-dimensional matrices: a computational study. Interface Focus. 2016;6(5):20160040.

[2] Cao X, Moeendarbary E, Isermann P, Davidson PM, Wang X, Chen MB, et al. A chemomechanical model for nuclear morphology and stresses during cell transendothelial migration. Biophys J. 2016;111(7):1541–1552.
