# Supplementary material for: Nuclear plasticity increases susceptibility to damage during confined migration
Source: PLoS Comput Biol. 2020 Oct 9;16(10):e1008300. doi: 10.1371/journal.pcbi.1008300 (PMC7577492; doi:10.1371/journal.pcbi.1008300)
Supplement: S2 Table — (PDF) [file pcbi.1008300.s003.pdf]

S2 Table. Material Parameters.

Component	Density (ρ) (kg/m^3)	Young's Modulus (E) (kPa)	Poisson's ratio (ν)	Reference
Cell Membrane	1050	0.3	0.3	
Cytoplasm	1030	0.001	0.3	[1]
Nuclear Membrane	1800	0.2 - 5	0.3	[2]
Nucleus	1800	0.2 - 5	0.3	[2, 3]
Tissue 1, 2	1500	0.13 - 5	0.3	[4]

References

[1] Guo M, Ehrlicher AJ, Mahammad S, Fabich H, Jensen MH, Moore JR, et al. The role of vimentin intermediate filaments in cortical and cytoplasmic mechanics. Biophys J. 2013;105(7):1562–1568.

[2] Dahl KN, Engler AJ, Pajerowski JD, Discher DE. Power-law rheology of isolated nuclei with deformation mapping of nuclear substructures. Biophys J. 2005;89(4):2855–2864.

[3] Stephens AD, Banigan EJ, Adam SA, Goldman RD, Marko JF. Chromatin and lamin A determine two different mechanical response regimes of the cell nucleus. Mol Biol Cell. 2017;28(14):1984–1996.

[4] George E, Barai A, Shirke P, Majumder A, Sen S. Engineering interfacial migration by collective tuning of adhesion anisotropy and stiffness. Acta Biomater. 2018;72:82–93.
