# Supplementary figures and images for: Nuclear plasticity increases susceptibility to damage during confined migration
Source: PLoS Comput Biol. 2020 Oct 9;16(10):e1008300. doi: 10.1371/journal.pcbi.1008300 (PMC7577492; doi:10.1371/journal.pcbi.1008300)

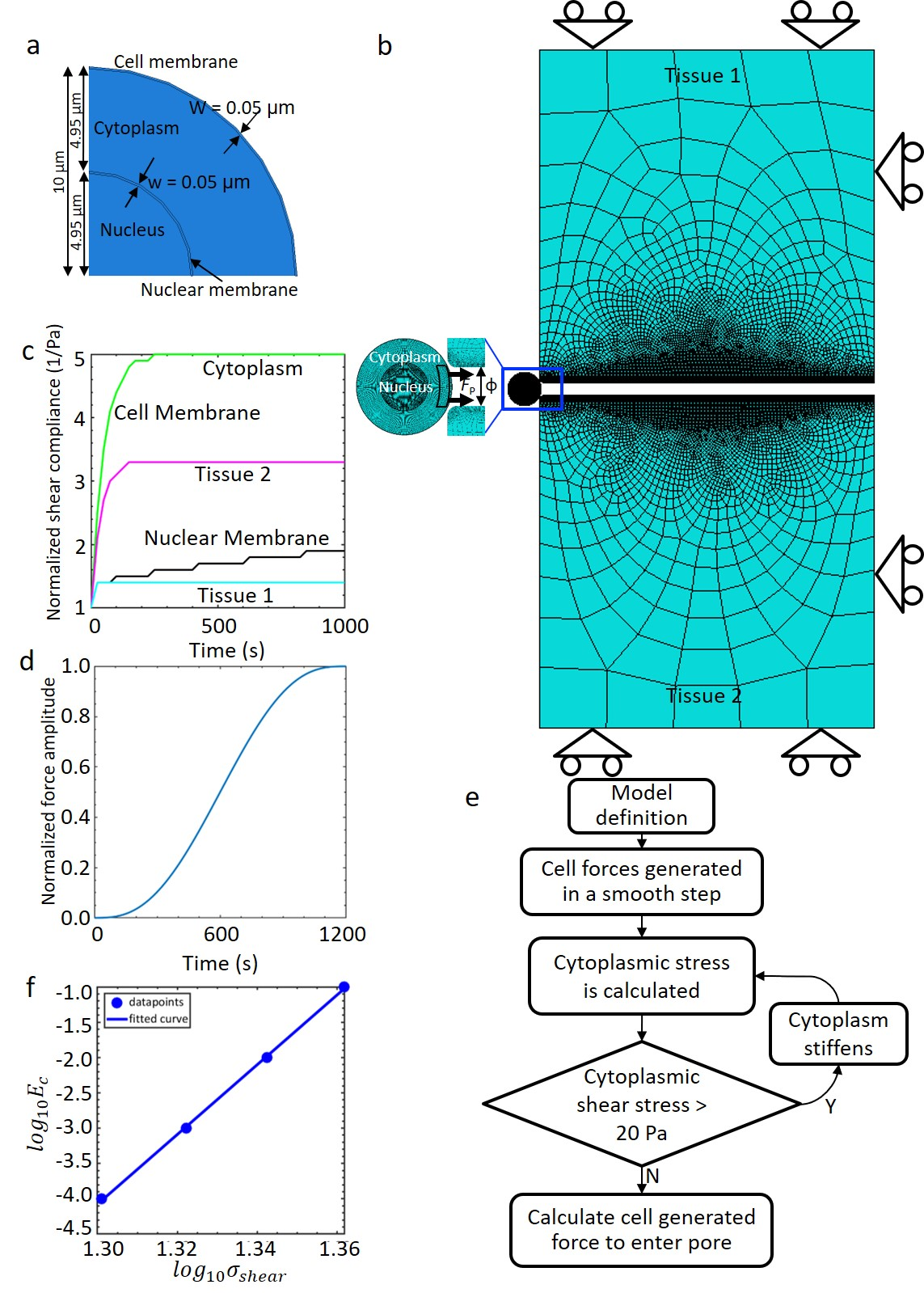

Supplement: S1 Fig — (a) Dimensions of various parts of the modelled cell. (b) Finite element model with mesh. Lateral and transverse boundaries of the tissue (1 and 2) are constrained in their perpendicular directions. (c) Viscoelastic properties of various materials in the model. (d) Temporal variation of input force. (e) Simulation process flow. (f) Assumed dependence of cytoplasmic stiffness (Ec) with shear stress (σshear) encountered by the cell. Ec is increased in discrete steps as indicated by datapoints and a smooth curve is interpolated, i.e., the points are used to define a function between the two variables. (TIF) [file pcbi.1008300.s004.tif]

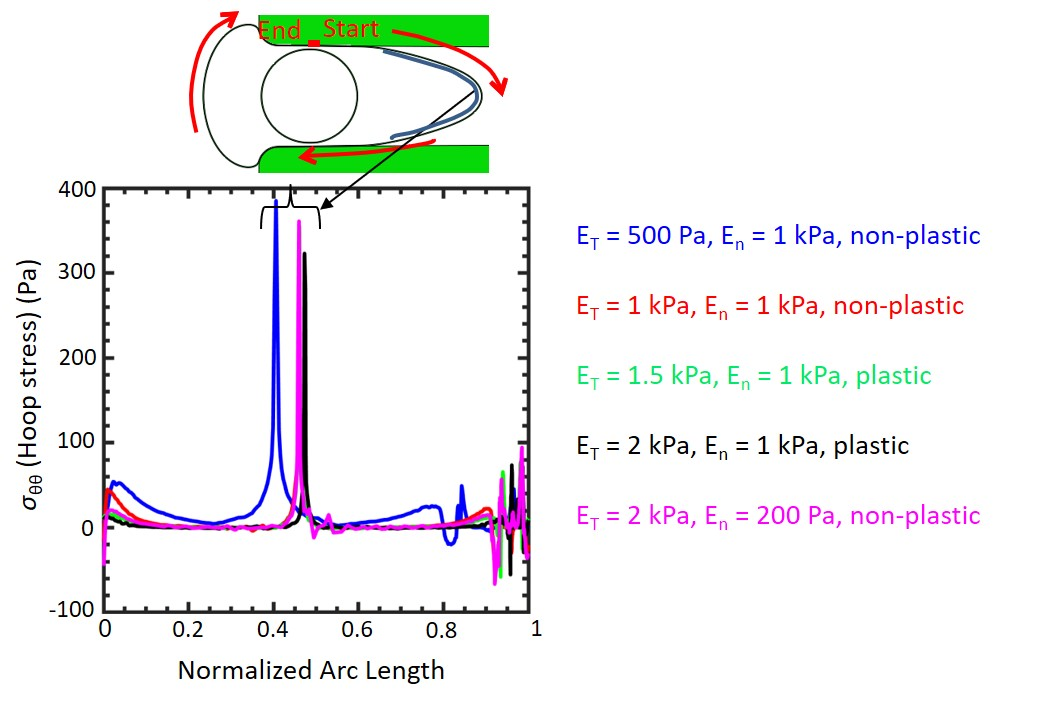

Supplement: S2 Fig — For a confinement of D0/ϕ = 1.67, the spatial variation of hoop stresses along the length of the membrane for different combinations of ET and En. (TIF) [file pcbi.1008300.s005.tif]

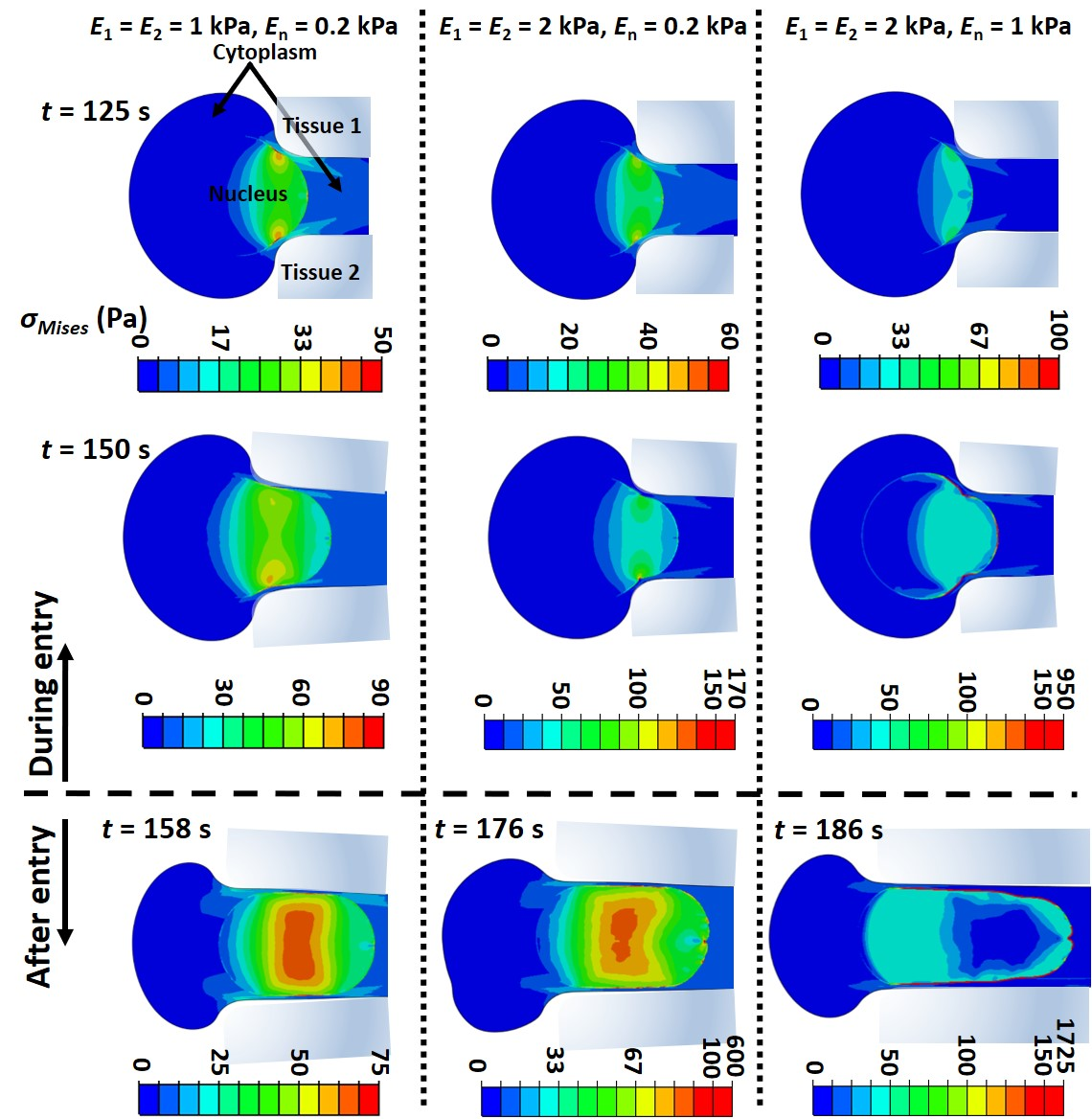

Supplement: S3 Fig — The spatiotemporal evolution of stress distribution during and just after entry of the 5μm nucleus into a 3μm pore, i.e., D0/ϕ = 1.67. Contours and colourbars indicate von Mises stresses (σMises) developed in the cytoplasm and nucleus. The cell membrane has not been displayed in the figures for clarity. (TIF) [file pcbi.1008300.s006.tif]

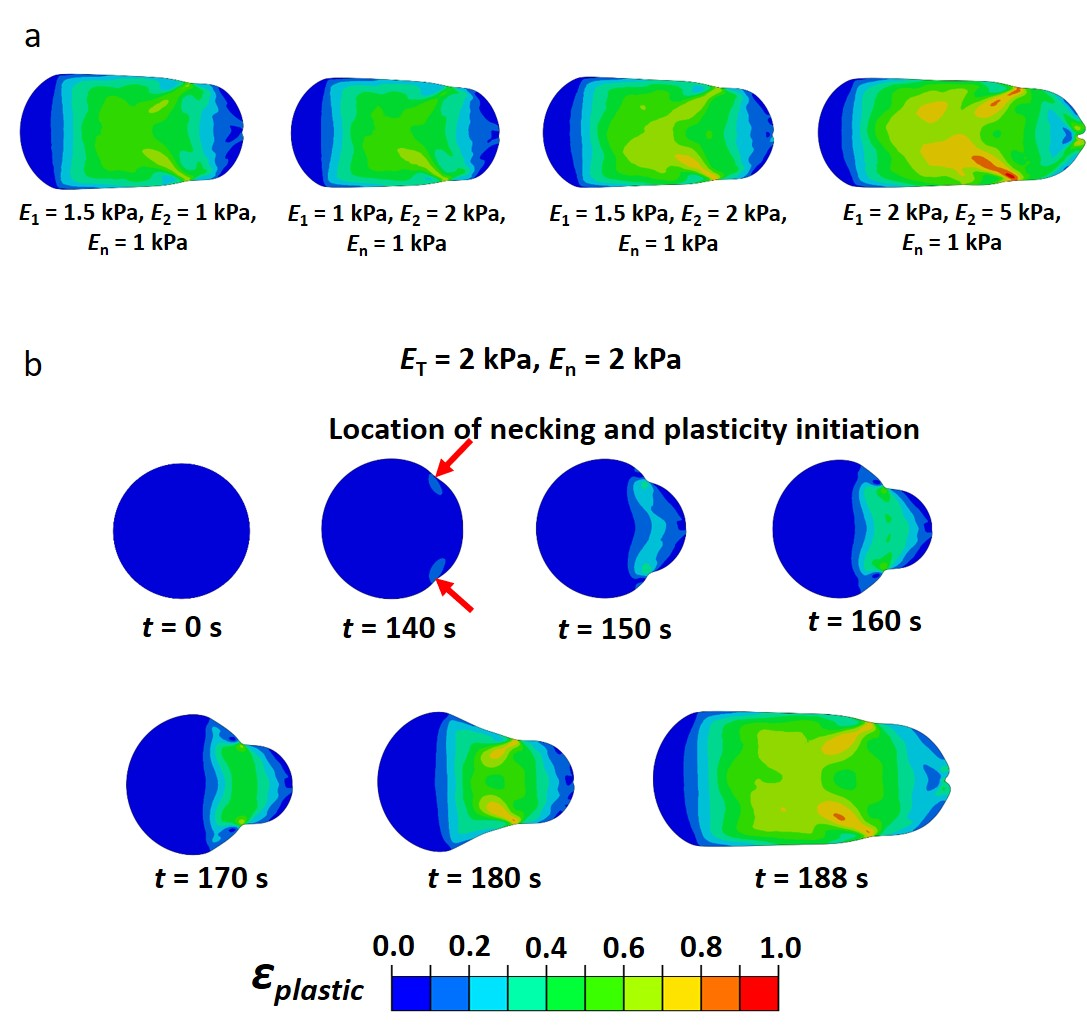

Supplement: S4 Fig — (a) E1 and E2 refer to the Young’s moduli of tissues 1 and 2 on both sides of the interface. D0/ϕ = 1.67 for all the cases. Contours represent the spatial distribution of plastic strain (εplastic). (b) Plastic strain accumulated in a cell as a function of time during constricted migration for D0/ϕ = 1.67. En = ET = 2 kPa. Red arrows indicate the region where necking first occurs and plasticity is initiated. The colourbar indicates magnitude of plastic strain in the nucleus (εplastic = εtotal − εelastic). (TIF) [file pcbi.1008300.s007.tif]

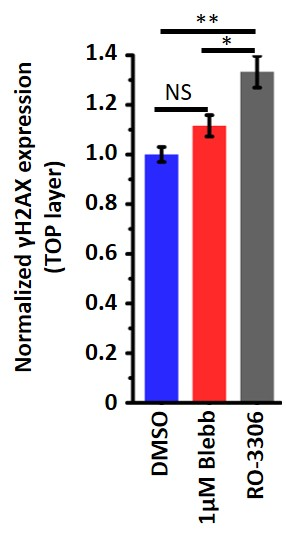

Supplement: S5 Fig — (TIF) [file pcbi.1008300.s008.tif]

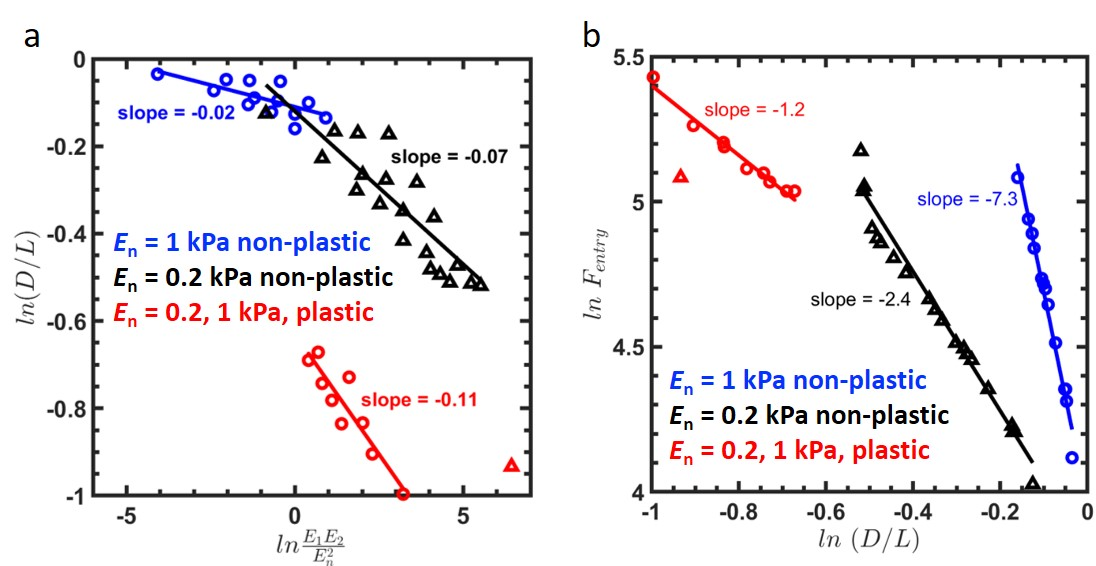

Supplement: S6 Fig — Scaling between nuclear circularity and (a) the coupled effect of tissue and nuclear stiffness, and (b) force required by a cell to enter a pore. All datapoints refer to the condition D0/ϕ = 1.67. E1 and E2 vary from 0.13 to 5 kPa. (TIF) [file pcbi.1008300.s009.tif]
